# Increased TNF-α/IFN-γ/IL-2 and Decreased TNF-α/IFN-γ Production by Central Memory T Cells Are Associated with Protective Responses against Bovine Tuberculosis Following BCG Vaccination

**DOI:** 10.3389/fimmu.2016.00421

**Published:** 2016-10-17

**Authors:** Mayara F. Maggioli, Mitchell V. Palmer, Tyler C. Thacker, Hans Martin Vordermeier, Jodi L. McGill, Adam O. Whelan, Michelle H. Larsen, William R. Jacobs, W. Ray Waters

**Affiliations:** ^1^Infectious Bacterial Diseases of Livestock Research Unit, National Animal Disease Center, Ames, IA, USA; ^2^Imbio, Department of Veterinary Pathology, College of Veterinary Medicine, Iowa State University, Ames, IA, USA; ^3^Tuberculosis Research Group, Animal and Plant Health Agency, Addlestone, UK; ^4^Department of Diagnostic Medicine and Pathology, College of Veterinary Medicine, Kansas State University, Manhattan, KS, USA; ^5^Defense Science and Technology Laboratory, Porton Down, Wiltshire, UK; ^6^Department of Microbiology and Immunology, Albert Einstein College of Medicine, Bronx, NY, USA

**Keywords:** polyfunctional T cells, central memory T cells, bovine tuberculosis, calf model

## Abstract

Central memory T cell (Tcm) and polyfunctional CD4 T cell responses contribute to vaccine-elicited protection with both human and bovine tuberculosis (TB); however, their combined role in protective immunity to TB is unclear. To address this question, we evaluated polyfunctional cytokine responses by CD4 T cell effector/memory populations from bacille Calmette–Guerin (BCG) vaccinated and non-vaccinated calves by flow cytometry prior to and after aerosol challenge with virulent *Mycobacterium bovis*. Polyfunctional cytokine expression patterns in the response by Tcm, effector memory, and effector T cell subsets were similar between BCG-vaccinated and *M. bovis*-infected calves, only differing in magnitude (i.e., infected > vaccinated). BCG vaccination, however, did alter the kinetics of the ensuing response to virulent *M. bovis* infection. Early after challenge (3 weeks post-infection), non-vaccinates had greater antigen-specific interferon-γ (IFN-γ)/tumor necrosis factor-α (TNF-α) and lesser IFN-γ/TNF-α/IL-2 responses by Tcm cells than did vaccinated animals. Importantly, these differences were also associated with mycobacterial burden upon necropsy. Polyfunctional responses to ESAT-6:CFP10 (antigens not synthesized by BCG strains) were detected in memory subsets, as well as in effector cells, as early as 3 weeks after challenge. These findings suggest that cell fate divergence may occur early after antigen priming in the response to bovine TB and that memory and effector T cells may expand concurrently during the initial phase of the immune response. In summary, robust IFN-γ/TNF-α response by Tcm cells is associated with greater mycobacterial burden, while IFN-γ/TNF-α/IL-2 response by Tcm cells are indicative of a protective response to bovine TB.

## Introduction

Bovine tuberculosis (TB) is a chronic bacterial infection affecting livestock, humans, and wildlife (1). *Mycobacterium bovis*, a member of the *Mycobacterium tuberculosis* complex, is the primary agent of TB in cattle, which causes substantial economic hardship for livestock producers. It is estimated that 50 million cattle are infected worldwide, costing approximately $3 billion annually to the livestock industry. In Great Britain alone, over 300,000 cattle were slaughtered from 2003 to 2013 in an attempt to control the disease at a cost to taxpayers of £500 million (2). In addition to the socioeconomic ramifications of the disease, *M. bovis* poses a significant public health threat. The World Health Organization classifies bovine TB as one of the seven most neglected zoonotic diseases and as such, the disease is particularly devastating in resource poor settings due to limited regulatory control, consumption of non-pasteurized milk/non-inspected meat, and co-morbidities affecting host susceptibility and disease severity (3). In addition to both animal and public health significance, *M. bovis* infection in cattle is an excellent model for human TB as adaptive immune responses and the ensuing immunopathogenesis are remarkably similar to that of *M. tuberculosis* infection in humans. Indeed, studies in cattle have been essential for the development of control strategies applicable to humans, such as the tuberculin skin test, interferon-γ (IFN-γ) release assays (IGRA), bacille Calmette–Guerin (BCG) vaccination, and approaches to differentiate infected from vaccinated individuals/animals (DIVA) [reviewed by Ref. (4)]. The study of human TB, including mouse and non-human primate models, has also led to considerable progress in the understanding and control of bovine TB.

Immunological memory is a primary feature of adaptive immunity and an essential goal of vaccination (5). In naïve hosts, effector and memory T cells are generated through developmental programing of naïve cells following an infection or antigen exposure. If infection is controlled, the vast majority of T cells generated during the expansion phase are eliminated and memory T cells remain, sometimes for a lifetime (6). Two major subsets of memory T cells (i.e., CD45RA^−^/CD45RO^+^) in humans and cattle are distinguishable based on expression of the lymphoid homing receptors CD62L and CCR7, reflecting important differences in function. Central memory T cells (Tcm) express CD62L and CCR7, are long-lived, and home to lymphoid tissues, while effector memory T cells (Tem) lack CCR7 and express minimal to no CD62L (6). In humans, Tcm cells exhibit elevated interleukin 2 (IL-2) production and proliferation potential, are long-lived, and able to generate heterogenic progeny capable of both terminal differentiation and self-renewal (7). Tcm cells are higher producers of effector molecules, such as IFN-γ, but exhibit low proliferation capability (6, 8, 9). In cattle, CD45RO^+^ CCR7^+^ memory cells in long-term cultures express high levels of CD62L (secondary lymphoid tissue homing receptor) and CD44 (homing cell adhesion molecule) and exhibit greater proliferation potential as compared to CD45RO^+^ CCR7^−^ memory cells (10). While bovine CD45RO^+^ CCR7^+^ CD62L^hi^ memory cells (Tcm) are elicited by *M. bovis* infection, their role in the response to protective BCG vaccination has not been evaluated. However, long-term cultured IFN-γ ELISPOT assays (so called, cultured IFN-γ ELISPOT) may be used as a surrogate of Tcm responses (8, 10–13). In cattle, cultured IFN-γ ELISPOT (i.e., 10- to 14-day culture followed by overnight recall stimulation) responses to BCG ± subunit vaccines positively predict vaccine efficacy and duration of vaccine-induced protection (14–17). As with BCG vaccination of humans, protection provided by BCG in cattle varies widely (18, 19). In humans, cultured IFN-γ ELISPOT responses are detected in spontaneously cured TB subjects in the absence of *ex vivo* responses (i.e., overnight recall stimulation) (20). In contrast to the cultured IFN-γ ELISPOT, *ex vivo* assays detect primarily effector and Tem responses as a result of the brief stimulation period (i.e., 16–24 h) and rapid cytokine production (21–23).

Polyfunctional T cells simultaneously produce two or more cytokines with IFN-γ, IL-2, and tumor necrosis factor-α (TNF-α) being the most commonly measured (24, 25). Associations between protection and vaccination-induced polyfunctional T cells have been mainly studied in small animal models (26–28). In humans, strong polyfunctional responses are detected in *M. tuberculosis*-infected individuals and are generally a sign of disease progression. Still, high IL-2 production is associated with a positive clinical status (e.g., latent or treated disease), while a strong IFN-γ/TNF-α response is associated with a failed response (i.e., active TB) (29). Human polyfunctional responses to vaccination both prior to TB exposure and in previously exposed individuals (i.e., latent infection) are extremely variable, much like BCG vaccination efficacy, and conflicting findings are reported (30, 31). Furthermore, the relative lack of efficacious TB vaccines for humans hinders the clear assessment of correlates of protection, including that of polyfunctional T cells (26, 32–35). In cattle, T cell polyfunctionality has only been measured upon *ex vivo* recall stimulation (21, 36). These studies found no association between polyfunctional responses measured before challenge and vaccine success. However, polyfunctional responses to infection were associated with increased pathology and poor disease outcome (36). Polyfunctional responses by long-term cultured cells for enrichment of Tcm responses have not been evaluated in spite of the fact that cultured IFN-γ ELISPOT is one of the most promising protection correlates in cattle (14–17). Likewise, the discrimination of cell phenotype involved in cytokine production under both cultured and *ex vivo* conditions may be necessary to identify specific correlates of vaccine efficacy, useful for the prioritization of vaccine candidates for costly biosafety level 3 (BSL-3) efficacy trials.

Early secretory antigenic target-6 (ESAT-6) and culture filtrate protein-10 (CFP-10) are co-secreted proteins encoded by the region of differentiation (RD)-1 of *M. tuberculosis* complex mycobacteria. Loss of the RD-1 region is the primary attenuating defect of BCG, and this region is absent in non-tuberculous mycobacterial (NTM) species (37–40). The differential expression of ESAT-6:CFP10 by tuberculous mycobacteria and their robust immunogenicity enables use of these antigens as a tool to discriminate *M. bovis*-infected from NTM-exposed or BCG-vaccinated animals (41–45). Antigen 85A (Ag85A) and protein TB10.4 are immunodominant antigens present in *M. tuberculosis* complex mycobacteria and BCG strains (1). *M. bovis* purified protein derivative (PPD) is a complex antigenic formulation, including many antigens also found in NTM. In the present study, the use of these antigens permitted assessment of both broad (PPD) and specific (TB10.4, Ag 85A) responses to vaccination and subsequent challenge, or exclusively to infection (ESAT-6:CFP10) (1).

Vaccine preparations included in the present study were BCG Danish (Statens Serum Institut, Copenhagen, Denmark, Strain 1331) and a cocktail of four BCG Danish (Strain 1331) deletion strains including BCG Δ*fdrA*, BCG Δ*leuCD* Δ*pks16*, BCG Δ*metA*, and BCG Δ*mmaA4*. BCG Danish is a widely used human pediatric vaccine to reduce human TB. Likewise, BCG Danish has been shown to reduce bovine TB disease burden in both experimental and field studies and has a well-established safety profile in cattle (1, 46, 47). The BCG Danish deletion derivatives used in this study are more attenuated and safer than the parental BCG strain in immunocompromised mice [(48, 49) and Berney-Meyer et al., unpublished data]. In immunocompetent mice, the BCG deletions Δ*fdr8*, Δ*mmaA4*, and Δ*pks16* each result in enhanced mycobacterial immunogenicity through enhanced cross-presentation of mycobacterial antigens (Δ*fdr8*), cytokine modulation (Δ*mmaA4*), and biofilm formation (Δ*pks16*), as compared to the parental BCG [(48) and Berney-Meyer et al., unpublished data]. BCG mutants, such as these, may also be used as vaccine vectors to promote epitope-specific responses (e.g., BCG Δ*pks12* for enhanced CD8 responses) (50). In the present study and as presented elsewhere (51), BCG Danish and the cocktail of BCG Danish deletions (hereafter called BCG mutants) were equally protective and induced similar IFN-γ- and Th17-associated cytokine responses, as evaluated in *ex vivo* assays for diagnostic purposes.

A better understanding of the cattle immune system may be beneficial to the understanding of both human and bovine TB, adding in the development of improved vaccine strategies in both species. Here, we investigated cytokine production (i.e., all combinations of IFN-γ, TNF-α, and IL-2) by flow cytometric analysis of memory T cell subsets in response to BCG vaccination and subsequent challenge with virulent *M. bovis* to determine how the elicited immune response correlated with infection outcome. Our findings indicate that early after infection, robust IFN-γ/TNF-α responses by Tcm are associated with greater mycobacterial burden, while IFN-γ/TNF-α/IL-2 responses by Tcm cells are indicative of a protective response.

## Materials and Methods

### Animal Use Ethics and Biosafety

All studies were approved by the National Animal Disease Center Animal Care and Use and Institutional Biosafety committees and performed under appropriate project licenses within the conditions of the Animal Welfare Act. All animals were housed in appropriate biological containment facilities at the National Animal Disease Center. Given the low dose challenge and relatively short duration of the studies, animals did not develop clinical signs of bovine TB necessitating palliative therapy such as cough, dyspnea, anorexia, or weight loss. Strict biosafety protocols were followed to protect personnel from exposure to *M. bovis* throughout the study, including BSL-3 containment upon initiation of *M. bovis* challenge in animal rooms and standard BSL-3 laboratory practices for handling *M. bovis* cultures and samples from *M. bovis*-infected animals.

### *Mycobacterium bovis* Vaccination and Challenge Procedures

Holstein steers were obtained from bovine TB-free herds in IA, USA, and housed in a BSL-3 facility at the National Animal Disease Center, Ames, IA, USA. For the first experiment (study 1), animals were experimentally infected with 10^4^ colony-forming units (cfu) of *M. bovis* 10-7428 (*n* = 8) by aerosol inoculation as described by (52). For the second study (study 2), calves were randomly assigned to the following treatment groups: no vaccination (*n* = 10), vaccination with *M. bovis* BCG Danish (Strain 1331; *n* = 9), and vaccination with a cocktail of four BCG Danish deletion strains including BCG *Δfdr8*, BCG *ΔleuCD Δpks16*, BCG *ΔmmaA4* (48), and BCG *ΔmetA* (49) (*n* = 10). Vaccines (10^6^ cfu, total dose) were administered subcutaneously at 2 weeks of age. Animals received *M. bovis* 10-7428 by aerosol (5 × 10^2^ cfu) at 3.5 months of age (i.e., 3 months after vaccination) as described by Palmer et al. (52).

### Mycobacterial Isolation and Assessment of Lesions

All calves were euthanized ~3.5 months after challenge with virulent *M. bovis* by intravenous administration of sodium pentobarbital. Tissues were examined for gross lesions and processed for microscopic analysis and isolation of *M. bovis*. Tissues collected included lung; liver; and mandibular, parotid, medial retropharyngeal, mediastinal, tracheobronchial, hepatic, and mesenteric lymph nodes. Lymph nodes were sectioned at 0.5-cm intervals and examined. Each lung lobe was sectioned at 0.5- to 1.0-cm intervals and examined separately. Lungs and lymph nodes (mediastinal and tracheobronchial) were evaluated using a semiquantitative gross pathology scoring system adapted from Vordermeier et al. (53). Tissues collected for microscopic analysis were fixed by immersion in 10% neutral buffered formalin. For microscopic examination, formalin-fixed tissues were processed by standard paraffin-embedment techniques, cut in 5-μm sections, and stained with hematoxylin and eosin. Adjacent sections from samples containing caseonecrotic granulomata suggestive of bovine TB were stained by the Ziehl–Neelsen technique for identification of acid-fast bacteria. Microscopic tuberculous lesions were staged (I–IV) based on a scoring system developed by Wangoo et al. (54).

### Long-Term and *Ex Vivo* Cell Culture

Peripheral blood mononuclear cells (PBMCs) were isolated from buffy coat fractions of blood collected by jugular venipuncture in 2× acid citrate dextrose solution, using density gradient centrifugation with Ficoll-Paque (Sigma, St. Louis, MO, USA). Complete RPMI medium for PBMC cell culture was RPMI 1640 (GIBCO, Grand Island) supplemented with 2-mM l-glutamine, 25-mM HEPES buffer, 100 U/ml penicillin, 0.1 mg/ml streptomycin, 1% non-essential amino acids (Sigma), 2% essential amino acids (Sigma), 1% sodium pyruvate (Sigma), 50-mM 2-mercaptoethanol (Sigma), and 10% (v/v) fetal bovine sera (FBS). Long-term cell cultures were generated by stimulating 2 × 10^6^/ml PBMC with a cocktail of *M. bovis* PPD (PPD, 5 μg/ml, Prionics Ag, Sclieren, Switzerland), Antigen85A (Ag85A – 1 μg/ml, LIONEX Diagnostics and Therapeutics GmbH, Braunschweig, Germany), TB10.4 (1 μg/ml, LIONEX Diagnostics and Therapeutics GmbH), and/or ESAT-6/CFP-10 (1 μg/ml, kind gift from Chris Minion, Iowa State University) in complete RPMI medium. Cells were cultured (2 × 10^6^cells/well, 1 ml/well) in 24-well flat-bottom microtiter plates (Nunc, Thermo Fisher, Waltham, MA, USA) at 39°C/5% CO_2_ for 13 days. The normal body temperature of cattle (*Bos taurus*) is 39°C and incubation of human blood at 39°C, as compared to 37°C, augments cytokine responses (55). Complete media containing human rIL-2 (Sigma, 10 U/ml) was used to replace media from the PBMC cultures at days 3 and 7. Fresh complete media without IL-2 was used at days 10 and 12.

At day 13, cultured cells were plated (2 × 10^6^ of cultured PBMC/well) into 96-well ELISPOT plates (Millipore, Watford, UK) and incubated in the presence of autologous antigen-presenting cells (APCs) and PPD (5 μg/ml), Ag85A/TB104 (1 μg/ml of each protein), ESAT-6:CFP10 (1 μg/ml), pokeweed mitogen (PWM, Sigma) (1 μg/ml), or medium alone. Autologous APCs were isolated by adherence incubating 1 × 10^5^ freshly isolated PBMC in complete medium at 39°C/5% CO_2_ for 90 min in 96-well plates. Non-adherent cells were discarded, and the adherent cells (APCs) were washed four times with warm RPMI 1640 media. Fresh complete media containing antigen and long-term cultured cells were then incubated for 20 h at 39°C/5% CO_2_. Long-term cultured cells were then incubated for 16 h at 39°C/5% CO_2_ with Brefeldin A (Sigma, 10 μg/ml) added at 4 h of culture.

For *ex vivo* culture, fresh PBMC isolated from buffy coat fractions of blood collected in 2× acid citrate dextrose solution were plated into 96-well plates (2 × 10^5^) and stimulated with PPD (5 μg/ml), Ag85A/TB10^4^ (1 μg/ml of each protein), ESAT-6:CFP10 (1 μg/ml), PWM (1 μg/ml), or medium alone for 16 h at 39°C/5% CO_2_ with Brefeldin A (Sigma, 10 μg/ml) added at 4 h of culture (56).

### Flow Cytometry

Following the appropriate culture duration, cells were pooled from individual animal replicates according to *in vitro* treatments (i.e., stimulation). Cells were stained as described by Whelan et al. (21) for assessment of bovine polyfunctional CD4 T cells and Maggioli et al. (10) for assessment of bovine memory/effector CD4 T cell subsets with antibodies listed in Table 1. Intracellular staining was performed following BD Perm/Wash instructions (BD Biosciences, San Jose, CA, USA). Flow cytometric analysis was performed with a BD LSR flow cytometer (BD Biosciences). Data were analyzed using FlowJo X (Tree Star Inc., San Carlos, CA, USA). A representative plot of the gating strategy is depicted in Figure S1 in Supplementary Material. A representative unstimulated condition response is depicted in Figure S2 in Supplementary Material.

**Table 1:** **Primary and secondary monoclonal antibodies and staining reagents**.

Reagent	Specificity, source	Fluorophore, source
ILA11	Bovine CD4, *Washington State University*	PE-Cy7, SouthernBiotech
ILA116	Bovine CD45RO, *Washington State University*	APC-Cy7, Life Technologies
7D12	Human CCR7 (CD197), BD Pharmingen	AF 350, Life Technologies or Spectral Red, SouthernBiotech
MCA1783-PE	Bovine IFN-γ, AbD Serotec	Not applicable
MCA2334-FITC	Bovine TNF-α, BD Pharmingen	Not applicable
AbD14386-DyLight649	Bovine IL-2, AbD Serotec	Not applicable
Live/dead staining	Pacific Blue, Life Technologies	Not applicable

### Statistical Analysis

Data were analyzed using analysis of variance followed by Sidak’s or Tukey’s multiple comparison test or Student’s *t*-test using GraphPAD Prism 6.0 (GraphPAD Software Inc., La Jolla, CA, USA).

## Results

### *M. bovis* Infection of Cattle Elicits Specific Polyfunctional Cytokine Production by CD4 T Cells in Long-Term and *Ex Vivo* Cultures

**Figure 1:**
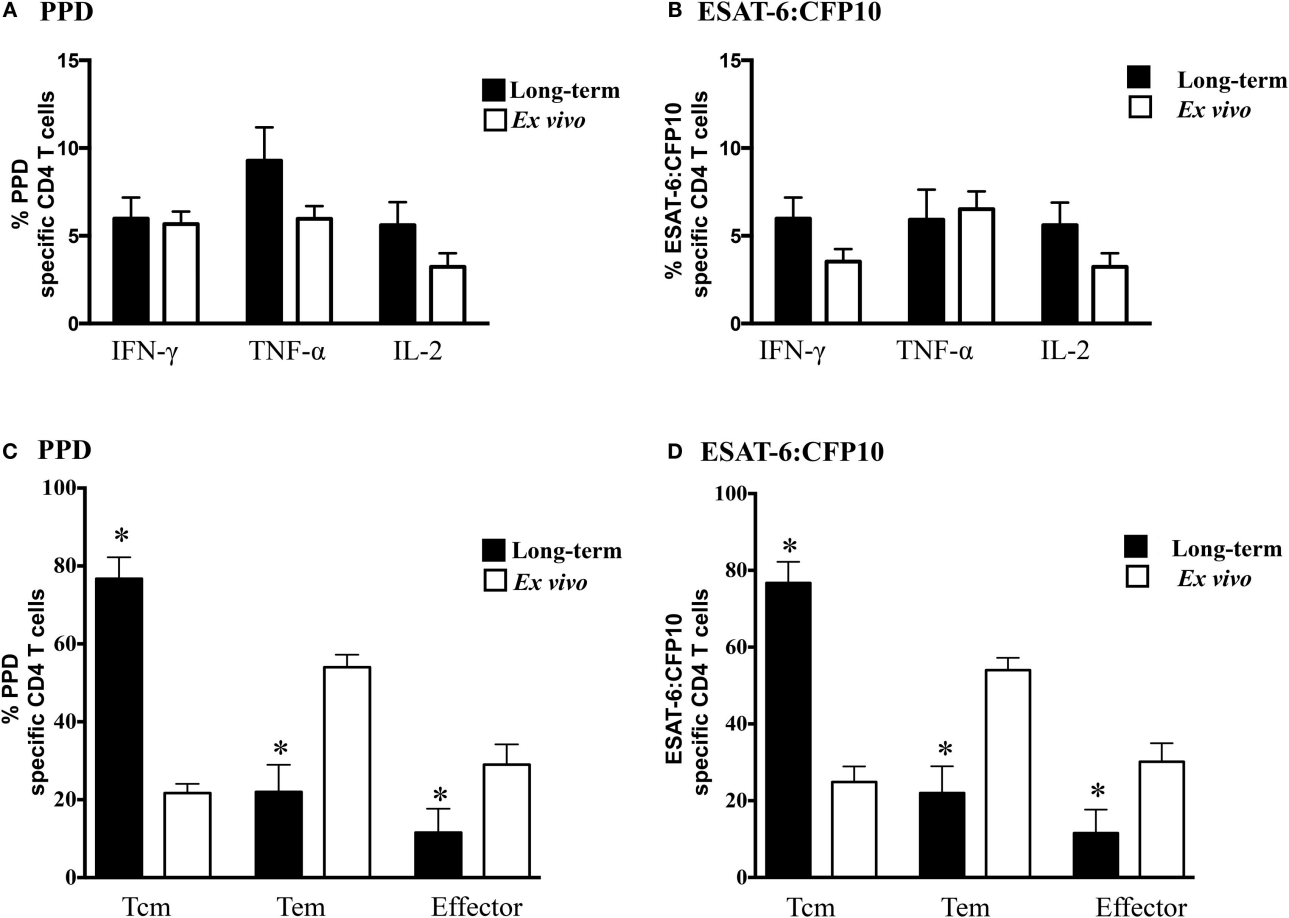
**Individual cytokines produced by different effector/memory phenotypes based upon culture conditions**. For long-term cultures, PBMCs from infected calves were isolated and stimulated with a cocktail of Ag85A, TB10.4, and ESAT-6:CFP10 as well as PPD for 13 days followed by transfer to 96-well round bottom plates with APCs and addition of media alone, PPD, or ESAT-6:CFP10 for an additional 16 h. For *ex vivo* culture, PBMCs were stimulated with media alone, PPD or ESAT-6:CFP10 for 16 h. Cytokine production and effector/memory phenotype were determined by ICS flow cytometry. Results are presented as average and SE for responses over the course of the study (*n* = 5 animals, 3 time points). Frequency long-term (closed bars) and *ex vivo* (open bars) cultured CD4 T cells producing IFN-γ, TNF-α, or IL-2 in response to PPD **(A)** or ESAT-6:CFP10 **(B)**. Phenotype of long-term (closed bars) and *ex vivo* (open bars) CD4 T cells producing any combination of IFN-γ, TNF-α, or IL-2 in response to PPD **(C)** or ESAT-6:CFP10 **(D)** based on CD45RO and CCR7 expression (Figure S1 in Supplementary Material). *Responses from PBMCs differ (*P* < 0.05, Sidak’s) between long-term and *ex vivo* cultures.

**Figure 2:**
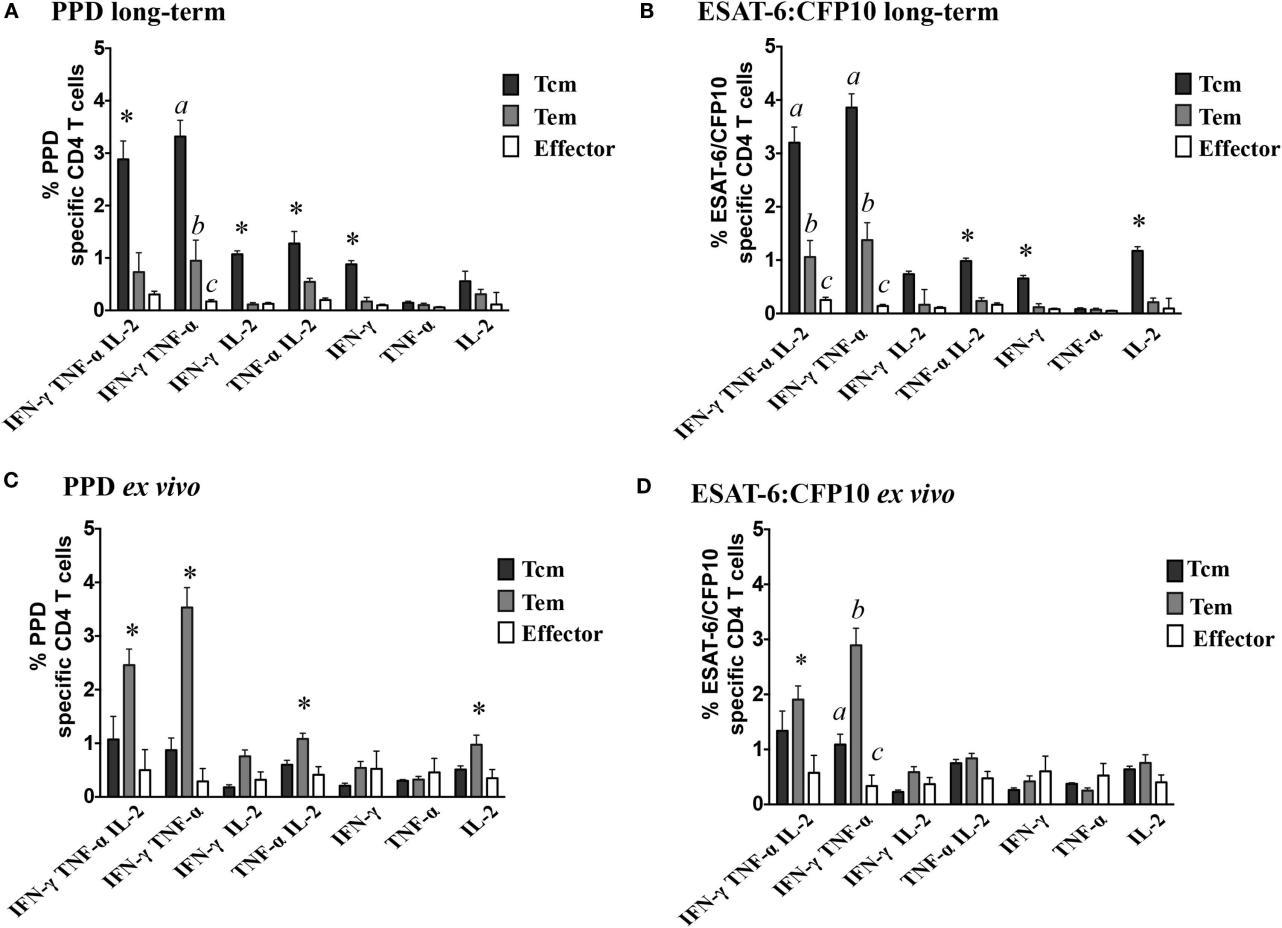
**Polyfunctional profiles by effector/memory phenotypes differ based on culture conditions**. For long-term culture, PBMCs were isolated and stimulated with a cocktail of Ag85A, TB10.4, and ESAT-6:CFP10 as well as PPD for 13 days followed by transfer to 96-well round bottom plates with APCs and addition of media alone, PPD, or ESAT-6:CFP10 for an additional 16 h. For *ex vivo* culture, PBMC were stimulated with media alone, PPD, or ESAT-6:CFP10 for 16 h. Cytokine production and effector/memory phenotype were determined by ICS flow cytometry. Results are presented as average and SE for responses over the course of the study (*n* = 30, 5 animals × 3 time points). Percentages of polyfunctional profiles in response to long-term stimulation with PPD **(A)** or ESAT-6:CFP10 **(B)** or *ex vivo* stimulation with PPD **(C)** or ESAT-6:CFP10 **(D)** split by effector/memory phenotype based on CD45RO and CCR7 expression (Figure S1 in Supplementary Material). *Responses differ (*P* < 0.05, Tukey’s) from the other two phenotypes. ^a,b,c^Different letters indicate differences (*P* < 0.05, Tukey’s) in effector/memory phenotypes within a particular polyfunctional subset.

Prior studies have demonstrated that *M. bovis* infection of cattle elicits polyfunctional CD4 T cell responses (21, 36); however, the relative contribution of effector, Tem, and Tcm subsets has not been evaluated. In the present study, individual cytokine (i.e., IFN-γ, TNF-α, and IL-2, Figures 1A,B) production by infected animals (study 1) did not differ between *ex vivo* or long-term cultured cells in response to either PPD or ESAT-6:CFP10 stimulation; however, the phenotype of responding cells greatly differed (*P* < 0.05) between culture conditions. In response to either PPD (Figure 1C) or ESAT-6:CFP10 (Figure 1D), Tcm cells (CD4^+^ CD45RO^+^ CCR7^+^) comprised ~80% of cytokine-producing cells in long-term cultures, while Tem (CD4^+^ CD45RO^+^ CCR7^−^) comprised ~60% of cytokine-producing cells in *ex vivo* cultures. Analysis of CD4 T cell cytokine profiles (i.e., all combinations of IFN-γ, TNF-α, and IL-2) revealed frequent coproduction of multiple cytokines under both *ex vivo* and long-term culture conditions in response to either PPD or ESAT-6:CFP10 by *M. bovis*-infected cattle (Table 2). IFN-γ/TNF-α and IFN-γ/TNF-α/IL-2 co-production were the predominant (*P* < 0.05) polyfunctional cytokine profile, regardless of culture condition. Also, long-term and *ex vivo* polyfunctional responses to either PPD or ESAT-6:CFP10 were comparable (i.e., comparison between the same profile under either *ex vivo* or long-term culture; *P* > 0.05) for all seven possible cytokine profiles (Table 2). As with the analysis of individual cytokine production, Tcm was the main phenotype contributing to polyfunctional responses in long-term cultures, whereas Tem was the main subset producing cytokines in *ex vivo* cultures (Figure 2).

**Table 2:** **IFN-γ/TNF-α/IL-2 and IFN-γ/TNF-α are the predominant polyfunctional profile in response to *M. bovis* infection, as assessed by long-term and *ex vivo* assays**.

**Cytokine profile^c^**	PPD		ESAT6:CFP10	
	Long-term Average (SEM)	*Ex vivo* Average (SEM)	Long-term Average (SEM)	*Ex vivo* Average (SEM)
IFN-γ/TNF-α/IL-2	3.92 (0.55)^a^	4.03 (0.78)^a^	4.51 (0.64)^a^	3.79 (0.71)^a^
IFN-γ/TNF-α	4.44 (0.51)^a^	4.69 (0.59)^a^	5.37 (0.59)^a^	4.32 (0.70)^a^
IFN-γ/IL-2	1.31 (0.09)^b^	1.26 (0.22)^b^	1.01 (0.36)^b^	1.18 (0.24)^b^
TNF-α/IL-2	2.02 (0.24)^b^	2.09 (0.24)^b^	1.39 (0.14)^b^	2.06 (0.30)^b^
IFN-γ	1.15 (0.12)^b^	1.27 (0.35)^b^	0.86 (0.13)^b^	1.28 (0.41)^b^
TNF-α	0.31 (0.05)^b^	1.08 (0.23)^b^	0.20 (0.06)^b^	1.15 (0.28)^b^
IL-2	0.98 (0.36)^b^	1.83 (0.29)^b^	1.48 (0.35)^b^	1.79 (0.34)^b^

*^a,b^Different letters indicate differences (*P* < 0.05) in the frequency of profiles within each culture condition (long-term or *ex vivo*). Differences in polyfunctional profile frequencies between long-term and *ex vivo* were not detected (*P* > 0.05, Tukey’s)*.

*^c^Data are presented as frequencies (%) of cytokine-producing cells within long-term or *ex vivo* cultures*.

### Vaccine Efficacy

**Figure 3:**
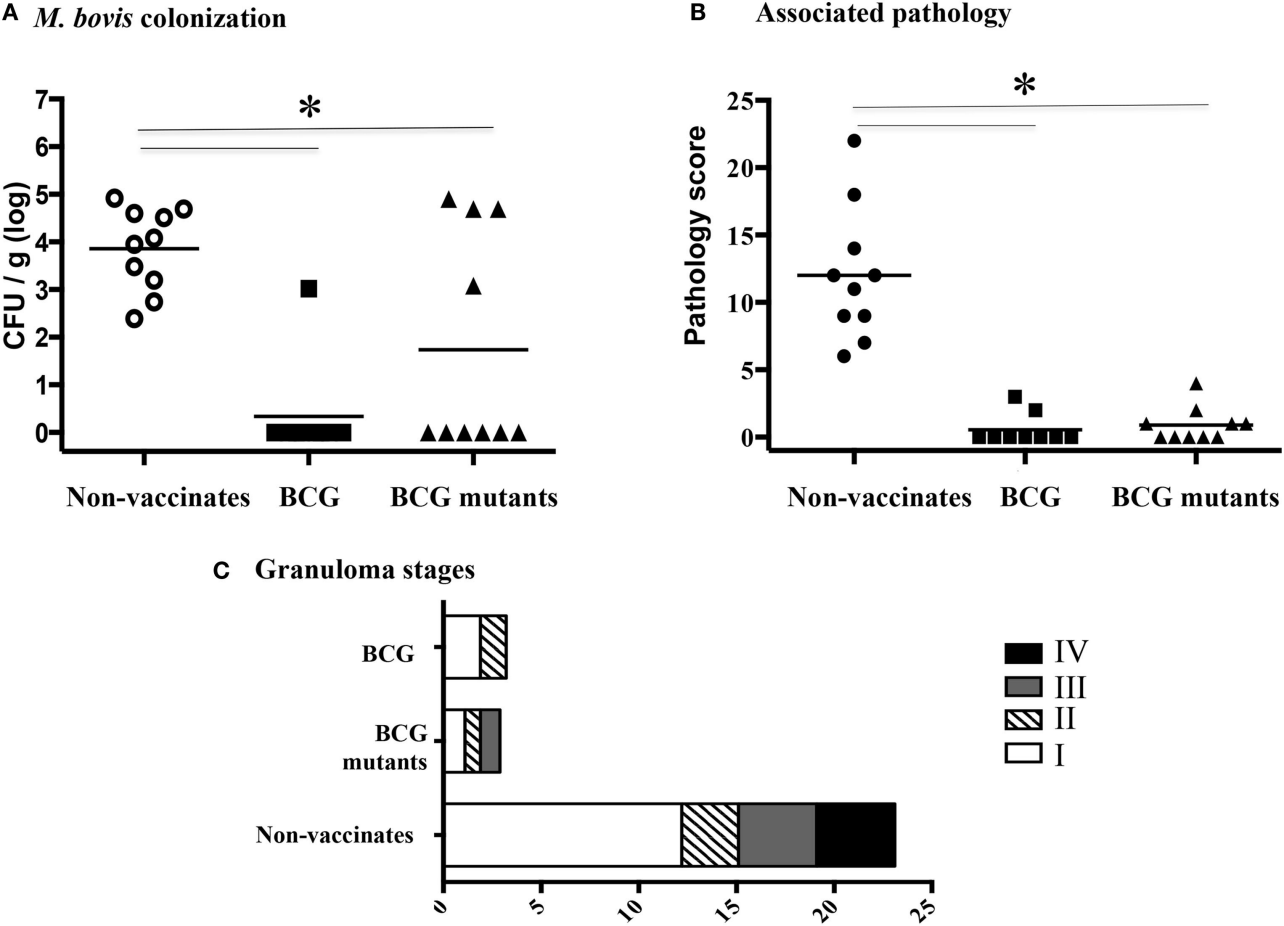
**BCG mutants and BCG vaccination confers protection against virulent *M. bovis* challenge**. **(A)** Quantitative assessment of mycobacterial burden in tracheobronchial lymph nodes. **(B)** Total gross pathology scores from lung and tracheobronchial and mediastinal lymph nodes. **(C)** Data are presented as average number of stages I–IV microscopic lesions observed in mediastinal lymph nodes, tracheobronchial lymph nodes, and lung based on the scoring system developed by Wangoo et al. (54). *Parameters differ (*P* < 0.05, Tukey’s) between non-vaccinates (*n* = 10) and BCG mutants (*n* = 10) or BCG vaccinates (*n* = 9).

Three and half months after vaccination (study 2), animals received virulent *M. bovis* by aerosol. Vaccine efficacy was assessed upon necropsy at 4.5 months after challenge. BCG mutants and BCG vaccinates had reduced bacterial load (Figure 3A, *P* < 0.05) and gross pathology as compared to non-vaccinates (Figure 3B, *P* < 0.05). Mean disease scores and *M. bovis* colonization did not differ between BCG mutants and BCG vaccinates. Lesion staging by histology corroborated gross lesion results (Figure 3C). Noteworthy, only non-vaccinated calves developed stage IV granulomas that generally contain large numbers of acid-fast bacilli (57), likely associated with increased transmission. In summary, both vaccines were exquisitely protective, decreasing pathology and bacterial burden.

### Cytokine Responses Elicited by BCG Vaccination

**Figure 4:**
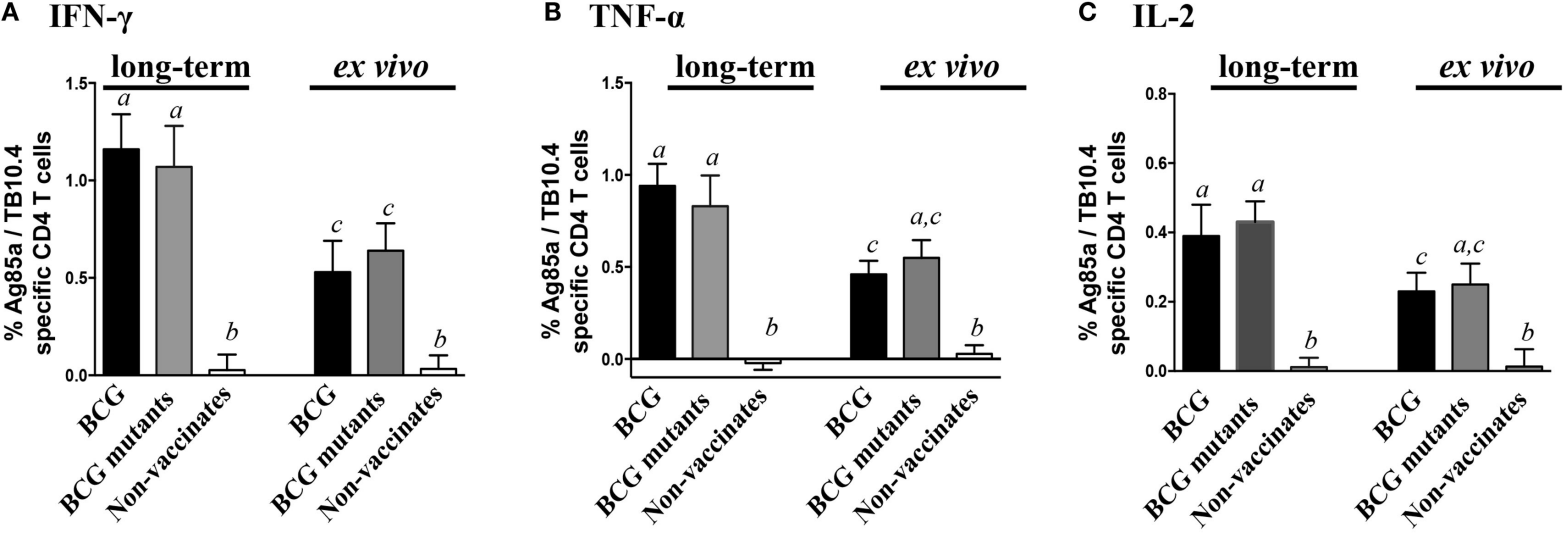
**Long-term and *ex vivo* cytokine responses to vaccination**. For long-term culture, PBMCs were isolated at 6 WPV and stimulated with a cocktail of Ag85A, TB10.4, and PPD for 13 days followed by transfer to 96-well round bottom plates with APCs and addition of media alone or Ag85A/TB10.4 for an additional 16 h. Results are presented as average and SE. Frequency of long-term (left) and *ex vivo* (right) cultured CD4 T cells producing IFN-γ **(A)**, TNF-α **(B)**, and IL-2 **(C)**. ^*a,b,c^Different letters indicate significant differences (*P* < 0.05) in cytokine production in long-term and *ex vivo* culture condition (Tukey’s).

**Figure 5:**
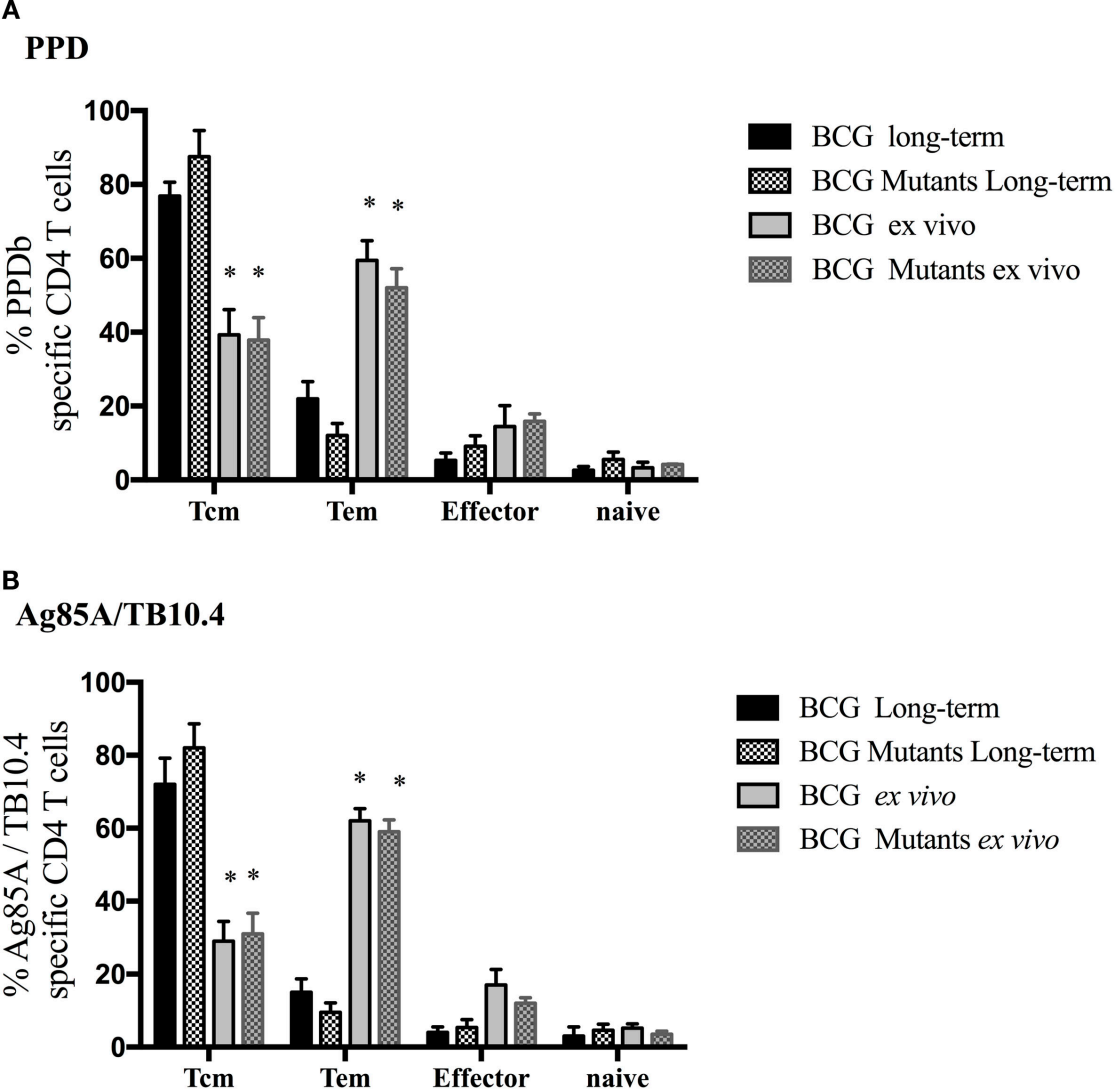
**Vaccine-elicited cytokine production is due to different effector/memory phenotypes based upon culture conditions**. For long-term culture, PBMCs were isolated at 6 WPV and stimulated with a cocktail of Ag85A, TB10.4, and PPD for 13 days followed by transfer to 96-well round bottom plates with APCs and addition of media alone PPD **(A)** or Ag85A/TB10.4 **(B)** for an additional 16 h. Cytokine production and effector/memory phenotype were determined by ICS flow cytometry. Results are presented as average and SE. Frequency of cytokine-producing cells under long-term or *ex vivo* culture exhibiting Tcm, Tem, effector, or naïve phenotype using the gating strategy described in Figure S1 in Supplementary Material. *Differences (*P* < 0.05) in cytokine production within memory subsets (i.e., Tcm, Tem, effector, or naïve) in long-term as compared to *ex vivo* cultures (Tukey’s).

**Figure 6:**
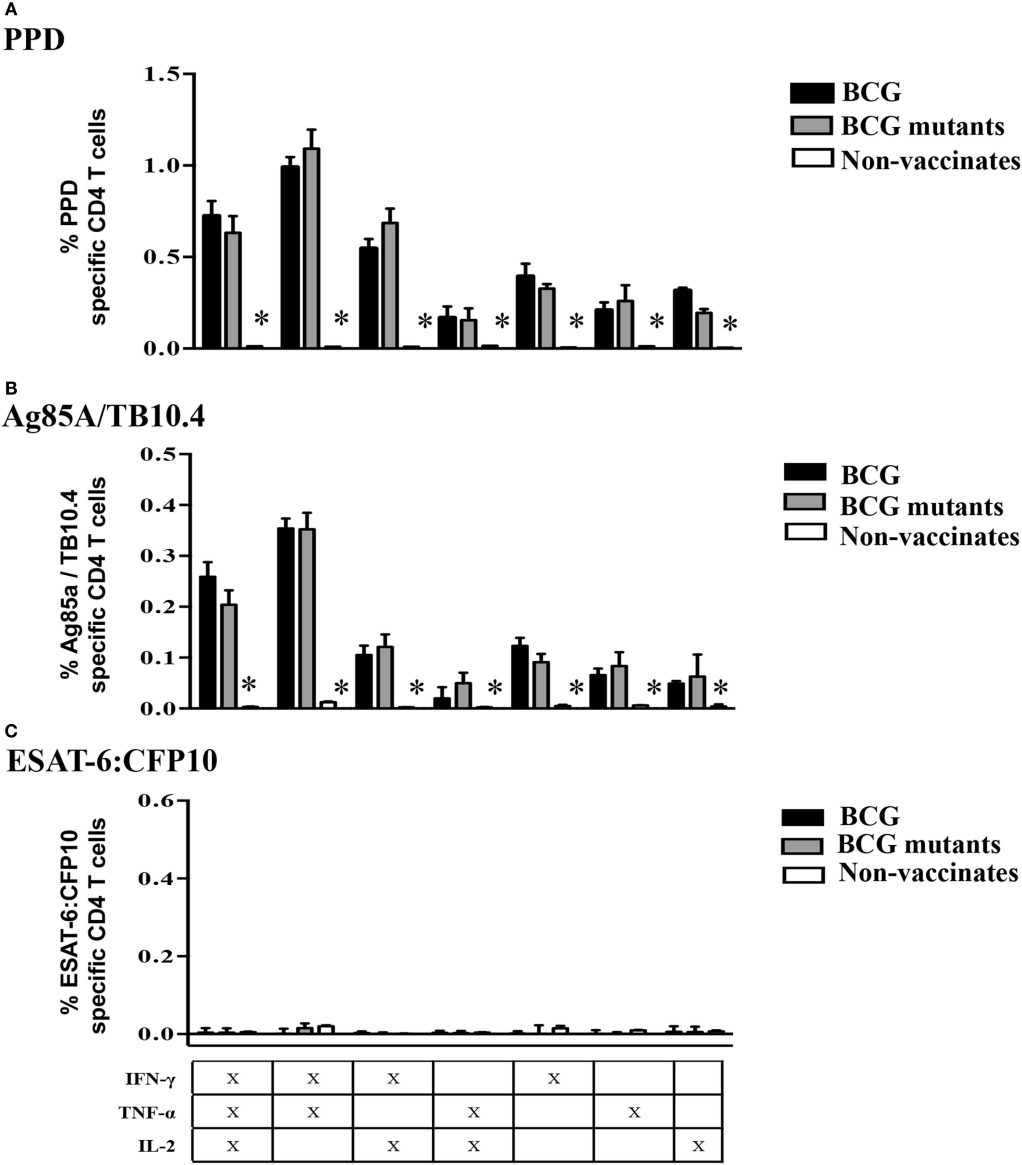
**Specific Tcm responses to vaccination at 6 weeks post-vaccination**. For long-term culture, PBMCs were isolated at 6 WPV and stimulated with a cocktail of Ag85A, TB10.4, and PPD for 13 days followed by transfer to 96-well round bottom plates with APCs and addition of media alone, PPD, Ag85A/TB10.4, or ESAT-6:CFP10 for an additional 16 h. Cytokine production and effector/memory phenotype were determined by ICS flow cytometry. Results are presented as average and SE. Polyfunctional responses by Tcm cells (gated on CD45RO^+^/CCR7^+^/CD4^+^ cells) were detected upon recall stimulation with PPD **(A)**, Ag85A/TB10.4 **(B)**, but not to ESAT-6:CFP10 **(C)**. *Different (*P* < 0.05, Tukey’s) responses between non-vaccinates and both vaccinated groups.

Vaccination of calves with either BCG or BCG mutants (study 2) elicited specific cytokine production at 6 weeks post-vaccination (WPV) in response to Ag85A/TB10.4 (Figure 4) and PPD (Figure S3 in Supplementary Material). As expected for this time point prior to *M. bovis* infection, non-vaccinated calves showed minimal or no responses to mycobacterial antigens. Long-term culture responses by vaccinated calves, as measured by IFN-γ, TNF-α, and IL-2, were generally higher (*P* < 0.05) than the respective *ex vivo* responses (Figure 4). As observed in infected calves (study 1), the proportion of Tcm, Tem, and effector cells contributing to cytokine production in response to PPD and Ag85A/TB10.4 differed between long-term and *ex vivo* cultures (Figure 5, *P* < 0.05). Responding cells in long-term cultures most frequently exhibited a Tcm phenotype, while *ex vivo* cytokine production was mainly due to Tem cells. The contribution of naïve cells to the cytokine response was minimal and did not differ based on vaccination or culture conditions. Cell phenotype (Tcm, Tem, and effector) and polyfunctional profiles within long-term or *ex vivo* cultures did not differ between BCG mutants and BCG vaccinates to Ag85A/TB10.4 (Figure S4 in Supplementary Material) nor PPD (Figure S5 in Supplementary Material) at 6 WPV. Vaccine-elicited polyfunctional responses were detected to PPD and Ag85A/TB10.4 but not to ESAT-6:CFP10 (Figure 6). Thus, BCG vaccination elicits a specific polyfunctional CD4 T cell response and the culture duration (long-term versus *ex vivo*) dictates whether the response is primarily within Tcm or Tem subsets, respectively. These findings also confirm that long-term cultured ELISPOT responses associated with protection to subsequent *M. bovis* infection in cattle are primarily a measure of CD4 Tcm responses (14, 58).

### Lower IFN-γ/TNF-α and Higher IFN-γ/TNF-α/IL-2 Coproduction by Tcms Early after Infection by Vaccinates as Compared to Non-Vaccinates

**Figure 7:**
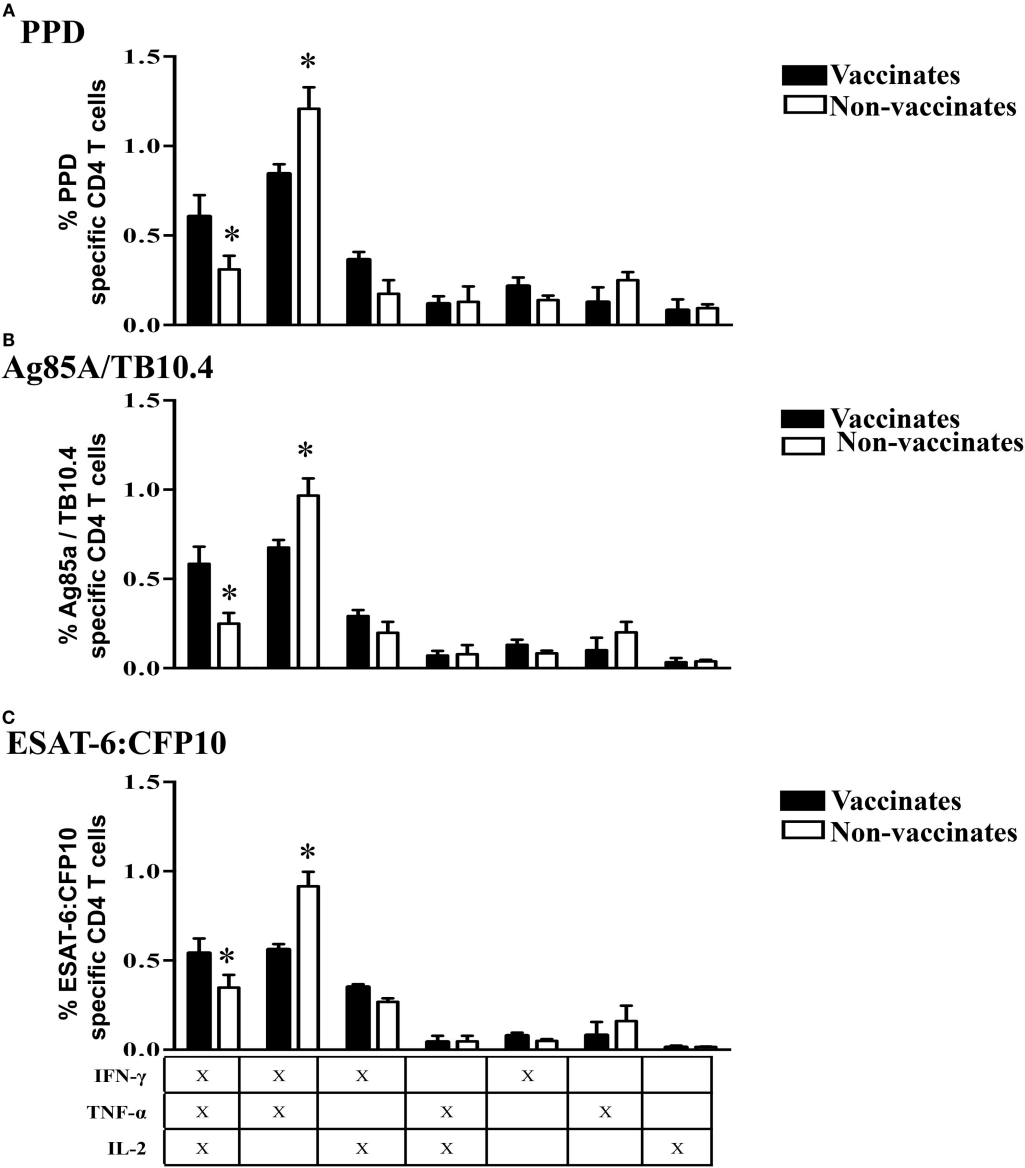
**Higher IFN-γ/TNF-α and lower IFN-γ/TNF-α/IL-2 Tcm responses by non-vaccinates versus vaccinates at 3 weeks post-challenge**. For long-term culture, PBMCs were isolated at 3 WPI and stimulated with a cocktail of Ag85A, TB10.4, and PPD for 13 days followed by transfer to 96-well round bottom plates with APCs and addition of media alone, PPD, Ag85A/TB10.4, or ESAT-6:CFP10 for an additional 16 h. Cytokine production and effector/memory phenotype were determined by ICS flow cytometry. Results are presented as average and SE. Polyfunctional responses by Tcm cells (gated on CD45RO^+^/CCR7^+^/CD4^+^ cells) were detected upon recall stimulation with PPD **(A)**, Ag85A/TB10.4 **(B)**, or ESAT-6:CFP10 **(C)**. *Parameters differ (*P* < 0.05, Tukey’s) in cytokine production by vaccinates versus non-vaccinates.

**Figure 8:**
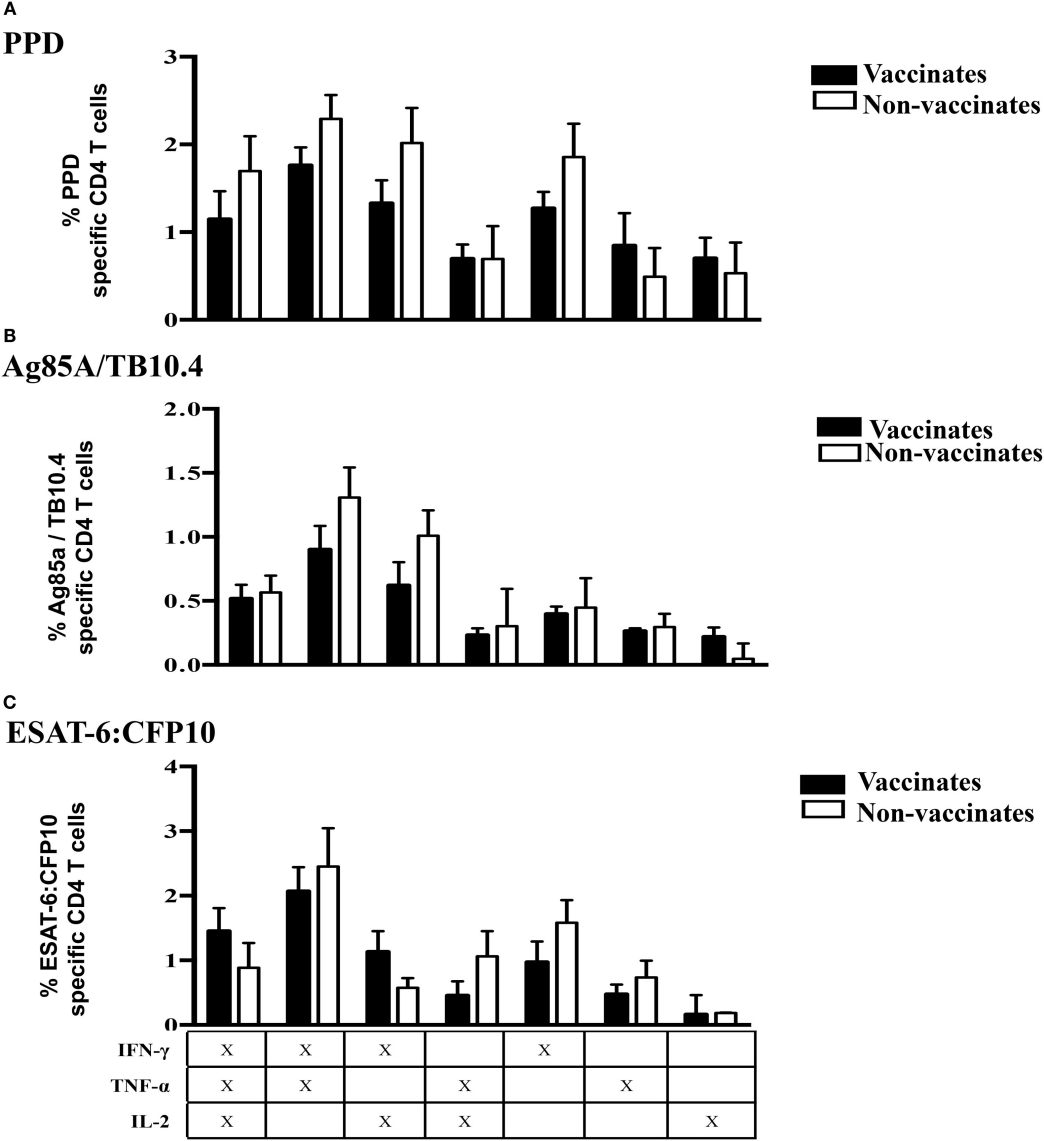
**By 8 weeks post-*M. bovis* challenge, polyfunctional responses by Tcm cells did not differ between vaccinates and non-vaccinates**. For long-term culture, PBMCs were isolated at 8 WPI and stimulated with a cocktail of Ag85A, TB10.4, and PPD for 13 days followed by transfer to 96-well round bottom plates with APCs and addition of media alone, PPD, Ag85A/TB10.4, or ESAT-6:CFP10 for an additional 16 h. Cytokine production and effector/memory phenotype were determined by ICS flow cytometry. Results are presented as average and SE. Polyfunctional responses by Tcm cells (gated on CD45RO^+^/CCR7^+^/CD4^+^ cells) upon recall stimulation with PPD **(A)**, Ag85A/TB10.4 **(B)**, or ESAT-6:CFP10 **(C)**. *Differences (*P* < 0.05, Tukey’s) in cytokine production by vaccinates versus non-vaccinates.

Early after challenge with virulent *M. bovis* [3 weeks post-infection (WPI)], non-vaccinates had greater (*P* < 0.05) IFN-γ/TNF-α responses to PPD, Ag85A/TB10.4, and ESAT-6:CFP10 by Tcm cells than did vaccinated animals (Figure 7). This difference was surprising given that vaccination elicited IFN-γ/TNF-α responses to PPD as well as Ag85A/TB10.4; and one might anticipate that these responses would be boosted after challenge with virulent *M. bovis*. In contrast, the IFN-γ/TNF-α/IL-2 response by vaccinates exceeded (*P* < 0.05) the respective response by non-vaccinates early after challenge (Figure 7). As with post-vaccination responses, responses by BCG mutants and BCG vaccinates did not differ at 3 WPI (Figure S5 in Supplementary Material) and 8 WPI (data not shown). Also, polyfunctional responses by Tcm cells at 8 WPI were similar among vaccinates and non-vaccinates (Figure 8). To investigate how vaccination and infection-elicited responses contrast, we compared the polyfunctional cytokine response by vaccinates at 6 WPV to that of non-vaccinates at 3 and 8 WPI. At 8 WPI, long-term and *ex vivo* responses to infection exceeded (*P* < 0.05) respective responses to vaccination for most polyfunctional profiles to both Ag85A/TB10.4 and PPD (Figure S7 in Supplementary Material). Importantly, both IFN-γ/TNF-α/IL-2 and IFN-γ/TNF-α responses to infection at 3 WPI (data not shown) and 8 WPI (Figure S7 in Supplementary Material) exceeded (*P* < 0.05) respective responses to vaccination upon recall stimulation of long-term cultures with Ag85A/TB10.4. Thus, both attenuated and virulent strains of *M. bovis* elicit polyfunctional T cell responses; however, these responses differ in magnitude.

**Figure 9:**
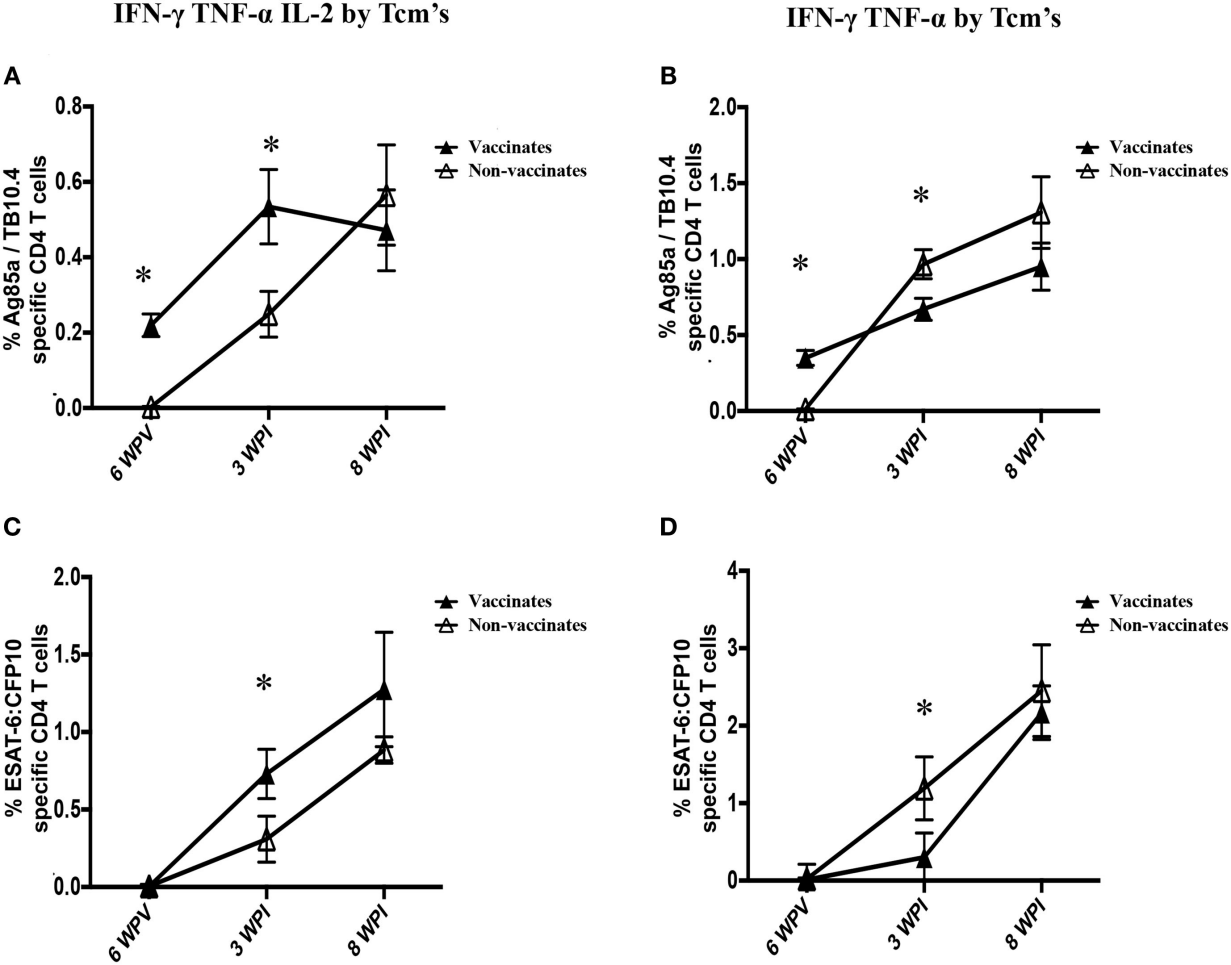
**Kinetics of IFN-γ TNF-α IL-2 and IFN-γ TNF-α responses by Tcm**. For long-term culture, PBMCs isolated at 6 weeks post-vaccination (6 WPV), 3 weeks post-infection (3 WPI) and 8 WPI were stimulated with a cocktail of Ag85A, TB10.4, and PPD for 13 days followed by transfer to 96-well round bottom plates with APCs and addition of media alone, Ag85A/TB10.4 or ESAT-6:CFP10 for an additional 16 h. Cytokine production and effector/memory phenotype were determined by ICS flow cytometry. Results are presented as average and SE. Percentages of long-term cultured Tcm cells (gated on CD45RO^+^/CCR7^+^/CD4^+^ cells) producing IFN-γ/TNF-α/IL-2 or IFN-γ/TNF-α upon recall stimulation with Ag85A/TB10.4 **(A,B)** or ESAT-6:CFP10 **(C,D)**. *Differences (*P* < 0.05, Tukey’s) in the cytokine production by non-vaccinates and vaccinates.

The most striking difference between vaccinates and non-vaccinates was the relative contribution of IFN-γ/TNF-α/IL-2 and TNF-α/IFN-γ production by Tcm cells early after challenge (3 WPI). The kinetics of long-term culture Tcm cells expressing these polyfunctional profiles is depicted in Figure 9. Vaccination elicited IFN-γ/TNF-α/IL-2 and IFN-γ/TNF-α polyfunctional Tcm responses to Ag85A/TB10.4 but not to ESAT-6:CFP10. Three weeks after challenge, IFN-γ/TNF-α responses by Tcm cells were greater in non-vaccinates as compared to vaccinates (Figure 9, *P* < 0.05), while vaccinates maintained higher percentages of IFN-γ/TNF-α/IL-2-producing Tcm cells in comparison to non-vaccinates (Figure 9, *P* < 0.05). At 8 weeks post-challenge, TNF-α/IFN-γ/IL-2 and TNF-α/IFN-γ responses were similar between vaccinates and non-vaccinates (Figure 9). Thus, BCG vaccination alters the ensuing polyfunctional profile upon infection with virulent *M. bovis*, favoring early IFN-γ/TNF-α/IL-2 production by CD4^+^ Tcm cells and dampening TNF-α/IFN-γ elicited by infection.

### Vaccination-Elicited Protection Is Associated with Reduced IFN-γ/TNF-α Responses by Tcm Cells Early after Challenge

**Figure 10:**
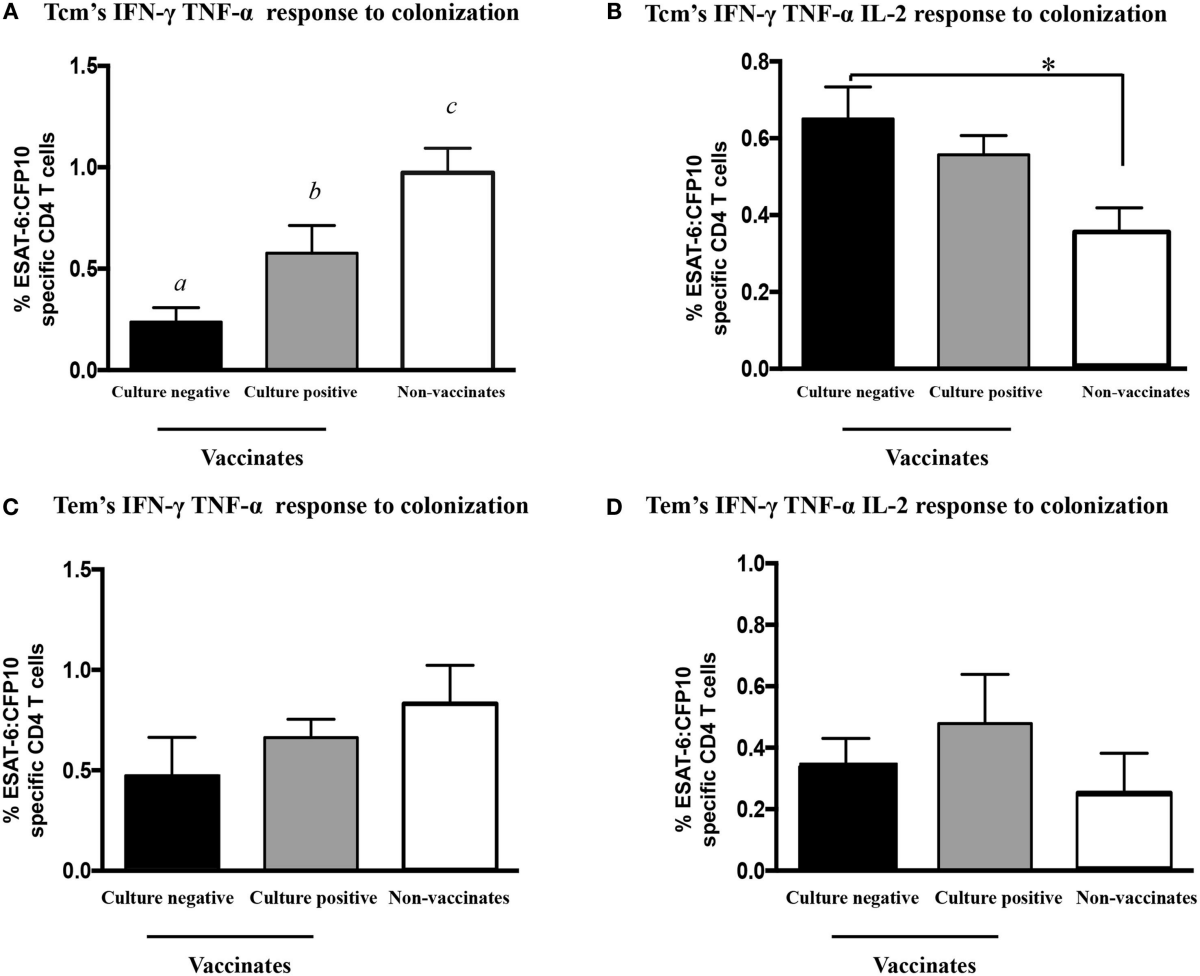
**IFN-γ/TNF-α/IL-2 and IFN-γ/TNF-α polyfunctional responses at 3 weeks post-infection by Tcm, but not by Tem cells, differ based upon *M. bovis* colonization and vaccination status**. Calves were grouped accordingly with both vaccine status and bacterial burden results (culture positive or negative) for assessment of IFN-γ/TNF-α/IL-2 and IFN-γ/TNF-α Tcm response by flow cytometry at 3 WPI. Long-term culture consisted of PBMCs isolated at 3 WPI and stimulated with a cocktail of Ag85A, TB10.4, and PPD for 13 days followed by transfer to 96-well round bottom plates with APCs and addition of media alone or ESAT-6:CFP10 for an additional 16 h. Results are presented as average and SE. IFN-γ/TNF-α and IFN-γ/TNF-α/IL-2 polyfunctional responses by Tcm cells (gated on CD45RO^+^/CCR7^+^/CD4^+^ cells) from vaccinates (either culture positive or negative) or non-vaccinates upon recall stimulation with ESAT-6:CFP10 **(A,B)**. IFN-γ/TNF-α and IFN-γ/TNF-α/IL-2 polyfunctional responses by Tem cells (gated on CD45RO^+^/CCR7^−^/CD4^+^ cells) vaccinates (either culture positive or negative) or non-vaccinates upon recall stimulation with ESAT-6:CFP10 **(C,D)**. ^a,b,c^Different letters indicate differences (*P* < 0.05, Tukey’s) in cytokine production among vaccinates (culture positive and negative) and non-vaccinates. *Differences (*P* < 0.05, Tukey’s) in the cytokine production between culture-negative vaccinates and non-vaccinates.

After *M. bovis* challenge, Th-1 responses to mycobacterial antigens generally correlate with TB-associated pathology and poor vaccine efficacy (15, 53, 58, 59). In our study, both vaccines provided a high level of protection. *M. bovis* was isolated from only five vaccinated animals (four BCG mutants and one BCG vaccinate). Retrospective analysis comparing responses to early infection (3 WPI) by non-vaccinates versus culture-negative vaccinates (*n* = 14) or culture-positive vaccinates (*n* = 5) revealed differential IFN-γ/TNF-α responses by Tcm cells to ESAT-6:CFP10 among the groups (Figure 10A, *P* < 0.05). IFN-γ/TNF-α responses by Tcm cells from non-vaccinates were higher than that of both *M. bovis* culture-negative and -positive vaccinates (Figure 10A, *P* > 0.05). *M. bovis* culture-positive vaccinates produced intermediate cytokine levels, while culture-negative animals had the lowest IFN-γ/TNF-α responses (Figure 10A, *P* < 0.05). Conversely, IFN-γ/TNF-α/IL-2 production by Tcm cells was higher in culture-negative vaccinates as compared to culture-positive non-vaccinates (Figure 10B, *P* > 0.05). IFN-γ/TNF-α/IL-2 responses by Tcm cells from culture-positive vaccinates were intermediate, not differing from either culture-negative vaccinates or non-vaccinates (Figure 10B, *P* < 0.05). IFN-γ/TNF-α/IL-2 and IFN-γ/TNF-α production by Tcm cells in *ex vivo* assays did not differ among culture-negative and culture-positive animals (Figures 10C,D, *P* > 0.05). Thus, these results indicate that strong IFN-γ/TNF-α responses by Tcm cells are associated with greater mycobacterial burden, while IFN-γ/TNF-α/IL-2 responses by Tcm cells are indicative of a protective response provided by vaccination.

## Discussion

Tuberculosis in cattle shares many features with TB in humans, and studies with neonatal calves are particularly pertinent to human TB, since children are the primary target population for vaccination with exposure to TB often occurring at a very young age (1). Also, in contrast to laboratory mice, cattle are out-bred; thus, experimental variance is more similar to that of humans and of relevance for evaluation of safety and efficacy of vaccines, adjuvants, or other administered biologics. This can have significant advantages for a translational understanding of the mechanisms of pathogenesis and, more importantly, immunity. While correlates of vaccine-induced protection against TB are not entirely understood, Tcm cells are presumed to be critical for protection in cattle and humans (1, 13, 18, 20, 29, 32). Likewise, the role of polyfunctional CD4 T cells in the immune response to TB is not well established. Here, we have shown that two protective BCG vaccine formulations elicited similar polyfunctional responses in vaccinated calves, and that early upon challenge with virulent *M. bovis*, responses by protected animals exhibited a different profile of response by Tcm cells as compared to that of unprotected animals. Protected vaccinates had a lower frequency of ESAT-6:CFP10-specific Tcm cells coproducing IFN-γ and TNF-α, suggesting that the incitement of strong Tcm IFN-γ/TNF-α responses early upon infection is related to a higher bacterial burden and inability of the host to control mycobacterial growth. On the other hand, higher frequencies of Tcm cells producing IFN-γ/TNF-α/IL-2 early after infection were associated with vaccine-elicited protection and bacterial arrest by the host.

Polyfunctional cytokine responses were detected upon antigenic stimulation of freshly isolated and long-term cultured PBMCs with no difference in the percentages of cytokine-producing cells based on culture condition. Still, the phenotype of responding cells greatly differed, with enriched numbers of responding Tcm cells in long-term culture and Tem cells in *ex vivo* cultures. This finding is consistent with previous research evaluating the phenotype of antigen-specific cytokine-producing cells in long-term cultures in humans (8, 11, 12) and cattle (10). As previously detected in naturally infected cattle, experimental infection-elicited polyfunctional responses biased toward IFN-γ/TNF-α and IFN-γ/TNF-α/IL-2 profiles (21). In response to vaccination, long-term cultured PBMC responses were generally higher than that of *ex vivo* stimulated PBMCs. Long-term culture has been used as a method to improve the sensitivity of TB diagnostic tests for humans (60, 61), so it is reasonable to speculate that long-term culture enriches low frequency antigen-specific cells, as with vaccination, consequently aiding in response detection.

As early as 3 WPI, IFN-γ/TNF-α responses by both Tcm and Tem cells (i.e., in long-term cultured and *ex vivo* assays, respectively) from non-vaccinates exceeded that of vaccinates. Yet, whether detection of Tcm responses in *in vitro* cultures so early upon infection was a consequence of *in vitro* conditions or whether antigen-specific cells, or their progeny, maintained their true phenotype throughout the *in vitro* culture requires further investigation. Studies have shown that the antigen specificity of responding cells in *ex vivo* and cultured ELISPOT assays do not necessarily correlate to each other with HIV (62), EBV (63), hepatitis C virus (11), or malaria infection (64, 65), demonstrating that the nature of the response by cytokine-producing cells may differ based on culture conditions. Likewise, it could be argued that the stimulation protocol might have induced *in vitro* priming of naïve T cells. *In vitro* priming, however, is unlikely due to the relatively short duration of the assay, absence of peptide antigen stimulation during the initial 13-day culture phase, no addition of cytokines apart from IL-2, and no enrichment of dendritic cells – all of which are required for *in vitro* priming (66). The absence of response to vaccine antigens by non-vaccinates prior to challenge further supports the notion that priming of naïve cells occurs *in vivo* and not as a consequence of long-term culture. Moreover, IFN-γ responses to vaccination in long-term cultures are associated with protection upon *M. bovis* challenge, indicating that the assessed *in vitro* response does hold a connection to the *in vivo* outcome.

Polyfunctional responses to ESAT-6:CFP10 (antigens not synthesized by BCG strains) were detected in effector, Tem, and Tcm populations from BCG vaccinates early after challenge (3 WPI). This finding is consistent with the early asymmetric division model of maturation, indicating memory and effector T cells expand concomitantly during the effector phase of the response and relatively early upon infection (67–72). In the mouse model, BCG elicited Tcm cells are detected as early as 20 days after vaccination and correlate with improved T cell recruitment to the lung. Also, the adoptive transfer of Tcm, but not Tem cells, is sufficient to confer protection (5). Laouar et al. (68) demonstrated the appearance of CD8 memory cells during the acute phase of a primary response to lymphocytic choriomeningitis virus as early as 8 days after infection. Likewise, prime-boost vaccination with viral-vectored vaccines expressing malarial antigens induce both *ex vivo* and cultured IFN-γ ELISPOT responses by 7 days after boost (65). It is important to note that antigen clearance appears to have a crucial role on the kinetics of the response, and pathogen clearance in chronic infections, such as TB (including *M. bovis* infection in calves), is not always achieved. Moreover, the very factors governing effector/memory T cell differentiation remain largely unknown. Whether memory cells arise (a) as direct progenies of effector cells [linear differentiation model (73–75)], (b) *via* a linear process dictated by a progressive loss of T cell differentiation potential (i.e., from naïve → effector → memory → terminally differentiated cells) dependent on antigenic stimulation and signaling milieu [decreasing potential model (76, 77)], or (c) as a separate lineage from naïve cells [divergent differentiation model (67, 69)] has long been debated. Still, the connection between naïve CD4 T cells and the various effector and memory fates these cells follow remains elusive, and a single unifying theory accounting for the diversity of CD4 T cell memory has not been discovered (78, 79). This fact suggests that various mechanisms of CD4 T cell memory generation may be differentially engaged depending on various factors (e.g., level of inflammation) and that these models are not necessarily mutually exclusive. Still, cell fate divergence (i.e., asymmetric division) early after priming has been reported by several authors (5, 65, 67–69) and is supported by the present findings, suggesting that memory generation begins prior to antigen clearance.

Early upon infection, vaccinates exhibited higher numbers of antigen-specific IFN-γ/TNF-α/IL-2 Tcm cells than did non-vaccinates, and such dissimilar responses between vaccinates and non-vaccinates early after challenge is associated with disease outcome. Polyfunctionality is often associated with less differentiated T cells (Tcm and Tem, as opposed to terminally differentiated T cells expressing killer cell lectin-like receptor 1 – KLRG1) and better quality of responses (32, 76, 79). Persistent or prolonged antigen stimulation is related to a progressive loss of central memory T cell pool, resulting in unfit immune responses by terminally differentiated, short-lived cells that produce single cytokine or loss of effector function all together (32, 80). The higher numbers of IFN-γ/TNF-α/IL-2 Tcm cells may indicate that vaccinated calves were able to control antigen load, retaining greater levels of cell functionality, which was beneficial in the long run. In future studies, we will evaluate KLRG1 expression on CD4 T cell effector/memory populations with the various polyfunctional profiles over a longer course of *M. bovis* infection to determine KLRG1 association with the expression of effector function by T cells in cattle.

As both vaccine treatments were proven to be exceptionally protective, we investigated whether vaccinated animals with no detectable *M. bovis* by quantitative culture responded to infection differently than did vaccinated animals with detectable *M. bovis* and/or non-vaccinates (all of which had detectable *M. bovis* by quantitative culture). Surprisingly, IFN-γ/TNF-α responses by Tcm cells differed among all three groups, suggesting colonization, and perhaps antigen load, could be associated with the number of IFN-γ/TNF-α antigen-specific cells. Intriguingly, similar numbers of Tcm cells producing IFN-γ/TNF-α/IL-2 were detected in vaccinates, regardless of their culture status. Still, IFN-γ/TNF-α/IL-2 responses were greater in vaccinates as compared to non-vaccinates, indicating that vaccine-induced IFN-γ/TNF-α/IL-2 responses may be associated with protection. Also, low level of TB-associated pathology was present in a few vaccinates with no detectable *M. bovis* by quantitative culture. It is known that few bacilli are required to cause TB-associated pathology, and the vaccine may have reduced the bacterial burden below the limit of *M. bovis* detection. Interestingly, three vaccinates with detectable *M. bovis* by quantitative culture showed no TB-associated pathology (lung and lung-associated lymph nodes). It is possible that TB-associated pathology was only delayed by vaccination, and these animals would still progress to clinical disease. It also indicates that even though vaccinates with detectable *M. bovis* had higher numbers of Tcm producing IFN-γ/TNF-α as compared to vaccinates with no *M. bovis*, the moderate levels of IFN-γ/TNF-α/IL-2 (or a combination of factors) would have conferred, if not a sterilizing protection, a significant decrease/delay in TB-associated pathology even in the presence of the pathogenic bacilli. This scenario would still be beneficial to the host.

Central memory T cells expressing IL-2 are associated with latent TB or effective anti-mycobacterial therapy in humans, whereas patients with active TB predominately exhibit effector cells that express IFN-γ/TNF-α (34, 81, 82). This suggests that CD4 T cell functional capacity is driven toward terminally differentiated T cells by bacterial load and continuous antigen exposure (34). While the occurrence of latency is not well established in cattle, non-vaccinates and non-protected vaccinates exhibited higher IFN-γ/TNF-α responses by Tcm cells than did protected calves early after challenge, similar to what occurs with active disease in humans. Additionally, protected vaccinates exhibited higher numbers of IFN-γ/TNF-α/IL-2-producing Tcm cells. It is plausible that the amount of initial antigen exposure controls the extent of differentiation and that vaccinated animals were more capable of containing the infection initially, preventing the loss of CD4 polyfunctional capacity. Moreover, single IL-2 producers did not differ among vaccinates and non-vaccinates or vary dependent upon *M. bovis* culture status at necropsy, which may indicate that the polyfunctional capacity, and not IL-2 production *per se*, was the most relevant protective response assessed. Such responses could be either a surrogate of other protective mechanisms or a just an indication of immune response fitness (e.g., the presence of Tcm exhibiting plasticity, more prone to long-term survival, or producing other cytokines). Protection to TB may require different levels of Tcm and Tem memory responses and also different ratios of Tcm to Tem in the different phases of this chronic infection (83). It is also possible that the presence of Tcm cells producing IFN-γ/TNF-α/IL-2 was a consequence of lower bacterial burden, rather than its cause. Importantly, Sakai et al. (84) demonstrated in the mouse model that differentiated T cells expressing KLRG1 elicited by BCG vaccination are retained within lung blood vasculature, lacking the ability to migrate into the lung parenchyma. Although these cells may produce high levels of IFN-γ, they are still functionally impaired and unable to confer protection in the lung. Kaushal et al. (85) showed that aerosol immunization of macaques with an *Mtb*Δ*sigH* mutant was highly protective against an otherwise lethal *M. tuberculosis* aerosol challenge. *Mtb*Δ*sigH* vaccinated macaques exhibited higher numbers of Tcm cells in response to vaccination (as compared with BCG or non-vaccinated controls) and higher percentages of IFN-γ/TNF-α/IL-2 in their lungs after challenge. The assessment of T cell survival, senescence, and peripheral migration markers [e.g., B-cell lymphoma 2 protein (BCL-2), KLRG-1, and programed cell death protein 1 (PD-1)], local responses in the lungs, as well the measure of a broader panel of cytokines would further the understanding of our findings, but such analyses were not achievable in the present experiments.

Predicting vaccine efficacy is difficult, in part, due to the lack of consistent immunological correlates of protection, especially one that would be appropriate throughout the different stages of chronic infections. Effective responses are likely not static, with different subsets displaying a variety of functions and migration capabilities necessary for disease prevention, bacterial arrest, and infection resolution. Recently, Ziraldo et al. (83) proposed a computational model to explore Tem and Tcm responses to a variety of mycobacterial antigens, generating a tool to predict vaccine formulations that would provide protective ratios of memory cells with desirable functionalities. If this approach proves to be successful, it would offer further insights into protection correlates and vaccination strategies to accomplish protection. In the meantime, present findings indicate that IFN-γ/TNF-α and IFN-γ/TNF-α/IL-2 responses by Tcm cells early after *M. bovis* infection in cattle are associated with detrimental and protective outcomes, respectively.

In summary, determining the T cell responses by measuring their functionality in combination with cell surface phenotype is likely to increase the fundamental understanding of T cell memory and effector differentiation, as these parameters define the T cell functional capacity, life-span potentials, antigen exposure history, and trafficking capabilities. Thus, phenotypic and functional analysis of T cells offers an assessment of the quality of the immune response, allowing a clearer evaluation of whether and how a response is protective, than either of these parameters measurement alone.

## Author Contributions

Conceived and designed the experiments: MP, TT, JM, WW, and MM. Performed the experiments: MM, MP, TT, and WW. Analyzed the data: MM and WW. Contributed reagents/materials/analysis tools: AW, HV, ML, and WJ. Wrote the paper: MM and WW.

## Disclaimer

USDA is an equal opportunity provider and employer. Mention of trade names or commercial products in this publication is solely for the purpose of providing specific information and does not imply recommendation or endorsement by the U.S. Department of Agriculture.

## Conflict of Interest Statement

The authors declare that the research was conducted in the absence of any commercial or financial relationships that could be construed as a potential conflict of interest.

## References

[1] Waters WR Palmer MV Buddle BM Vordermeier HM Bovine tuberculosis research: historical perspective and recent advances. Vaccine (2012) 30:2611–22. 10.1016/j.vaccine.2012.02.018 22342705

[2] Vordermeier HM Jones GJ Buddle BM Hewinson RG Villarreal-Ramos B. Bovine tuberculosis in cattle: vaccines, DIVA tests, and host biomarker discovery. Annu Rev Anim Biosci (2016) 4:87–109. 10.1146/annurev-animal-021815-111311 26884103

[3] WHO. The control of neglected zoonotic diseases: a route to poverty alleviation. Report of a Joint WHO/DFID-AHP Meeting. Geneva, Switzerland: WHO Headquarters (2006). Available from: http://www.who.int/zoonoses/Report_Sept06.pdf

[4] Waters WR Maggioli MF McGill JL Lyashchenko KP Palmer MV. Relevance of bovine tuberculosis research to the understanding of human disease: historical perspectives, approaches, and immunologic mechanisms. Vet Immunol Immunopathol (2014) 159:113–32. 10.1016/j.vetimm.2014.02.009 24636301

[5] Vogelzang A Perdomo C Zedler U Kuhlmann S Hurwitz R Gengenbacher M Central memory CD4+ T cells are responsible for the recombinant bacillus Calmette-Guérin δureC::hly vaccine’s superior protection against tuberculosis. J Infect Dis (2014) 210:1928–37. 10.1093/infdis/jiu347 24943726 PMC4241943

[6] Sallusto F Lanzavecchia A Araki K Ahmed R. From vaccines to memory and back. Immunity (2010) 33:451–63. 10.1016/j.immuni.2010.10.008 21029957 PMC3760154

[7] Farber DL Yudanin NA Restifo NP. Human memory T cells: generation, compartmentalization and homeostasis. Nat Rev Immunol (2014) 14:24–35. 10.1038/nri3567 24336101 PMC4032067

[8] Reece WHH Pinder M Gothard PK Milligan P Bojang K Doherty T A CD4(+) T-cell immune response to a conserved epitope in the circumsporozoite protein correlates with protection from natural *Plasmodium falciparum* infection and disease. Nat Med (2004) 10:406–10. 10.1038/nm1009 15034567

[9] Champagne P Ogg GS King AS Knabenhans C Ellefsen K Nobile M Skewed maturation of memory HIV-specific CD8 T lymphocytes. Nature (2001) 410:106–11. 10.1038/35065118 11242051

[10] Maggioli MF Palmer MV Thacker TC Vordermeier HM Waters WR Characterization of effector and memory T cell subsets in the immune response to bovine tuberculosis in cattle. PLoS One (2014) 10:e0122571 10.1371/journal.pone.0122571 PMC4400046 25879774

[11] Godkin AJ Thomas HC Openshaw PJ. Evolution of epitope-specific memory CD4(+) T cells after clearance of hepatitis C virus. J Immunol (2002) 169:2210–4. 10.4049/jimmunol.169.4.2210 12165552

[12] Todryk SM Bejon P Mwangi T Plebanski M Urban B Marsh K Correlation of memory T cell responses against TRAP with protection from clinical malaria, and CD4 CD25 high T cells with susceptibility in Kenyans. PLoS One (2008) 6:e2027. 10.1371/journal.pone.0002027 18446217 PMC2323567

[13] Blunt L Hogarth PJ Kaveh DA Webb P Villarreal-Ramos B Vordermeier HM Phenotypic characterization of bovine memory cells responding to mycobacteria in IFN-γ enzyme linked immunospot assays. Vaccine (2015) 33:7276–82. 10.1016/j.vaccine.2015.10.113 26549366

[14] Vordermeier HM Villarreal-Ramos B Cockle PJ McAulay M Rhodes SG Thacker T Viral booster vaccines improve *Mycobacterium bovis* BCG-induced protection against bovine tuberculosis. Infect Immun (2009) 77:3364–73. 10.1128/IAI.00287-09 19487476 PMC2715681

[15] Waters WR Palmer MV Nonnecke BJ Thacker TC Scherer CF Estes DM Efficacy and immunogenicity of *Mycobacterium bovis* DeltaRD1 against aerosol *M*. *bovis* infection in neonatal calves. Vaccine (2009) 27:1201–9. 10.1016/j.vaccine.2008.12.018 19135497 PMC2750035

[16] Whelan AO Wright DC Chambers MA Singh M Hewinson RG Vordermeier HM. Evidence for enhanced central memory priming by live *Mycobacterium bovis* BCG vaccine in comparison with killed BCG formulations. Vaccine (2008) 26:166–73. 10.1016/j.vaccine.2007.11.005 18055073

[17] Thom ML McAulay M Vordermeier HM Clifford D Hewinson RG Villarreal-Ramos B Duration of immunity against *Mycobacterium bovis* following neonatal vaccination with bacillus Calmette-Guérin Danish: significant protection against infection at 12, but not 24, months. Clin Vaccine Immunol (2012) 19:1254–60. 10.1128/CVI.00301-12 22718125 PMC3416081

[18] Black GF Weir RE Floyd S Bliss L Warndorff DK Crampin AC BCG-induced increase in interferon-gamma response to mycobacterial antigens and efficacy of BCG vaccination in Malawi and the UK: two randomised controlled studies. Lancet (2002) 359:1393–401. 10.1016/S0140-6736(02)08353-8 11978337

[19] Waters WR Palmer MV Thacker TC Davis WC Sreevatsan S Coussens P Tuberculosis immunity: opportunities from studies with cattle. Clin Dev Immunol (2011) 2011:768542. 10.1155/2011/768542 21197095 PMC3004413

[20] Millington KA Gooding S Hinks TSC Reynolds DJM Lalvani A. *Mycobacterium tuberculosis*-specific cellular immune profiles suggest bacillary persistence decades after spontaneous cure in untreated tuberculosis. J Infect Dis (2010) 202:1685–9. 10.1086/656772 20958211

[21] Whelan AO Villarreal-Ramos B Vordermeier HM Hogarth PJ. Development of an antibody to bovine IL-2 reveals multifunctional CD4 T(EM) cells in cattle naturally infected with bovine tuberculosis. PLoS One (2011) 6:e29194. 10.1371/journal.pone.0029194 22216206 PMC3245252

[22] Streitz M Tesfa L Yildirim V Yahyazadeh A Ulrichs T Lenkei R Loss of receptor on tuberculin-reactive T-cells marks active pulmonary tuberculosis. PLoS One (2007) 2:e735. 10.1371/journal.pone.0000735 17710135 PMC1936433

[23] Soares AP Scriba TJ Joseph S Harbacheuski R Murray RA Gelderbloem SJ Bacillus Calmette-Guérin vaccination of human newborns induces T cells with complex cytokine and phenotypic profiles. J Immunol (2008) 180:3569–77. 10.4049/jimmunol.180.5.3569 18292584 PMC2842001

[24] Caccamo N Dieli F Are polyfunctional cells protective in *M. tuberculosis* infection? In: Cardona PJ, editor. Understanding Tuberculosis – Analyzing the Origin of Mycobacterium tuberculosis Pathogenicity. (2012). Available from: http://www.intechopen.com/books/understanding-tuberculosis-analyzing-the-origin-of-mycobacterium-tuberculosis-pathogenicity/are-polyfunctional-cells-protective-in-m-tuberculosis-infection-

[25] Wilkinson KA Wilkinson RJ. Polyfunctional T cells in human tuberculosis. Eur J Immunol (2010) 40:2139–42. 10.1002/eji.201040731 20853500

[26] Aagaard C Hoang TT Izzo A Billeskov R Troudt J Arnett K Protection and polyfunctional T cells induced by Ag85B-TB10.4/IC31 against *Mycobacterium tuberculosis* is highly dependent on the antigen dose. PLoS One (2009) 4:e5930. 10.1371/journal.pone.0005930 19529771 PMC2691953

[27] Cruz A Torrado E Carmona J Fraga AG Costa P Rodrigues F BCG vaccination-induced long-lasting control of *Mycobacterium tuberculosis* correlates with the accumulation of a novel population of CD4^+^IL-17^+^TNF^+^IL-2^+^ T cells. Vaccine (2015) 33:85–91. 10.1016/j.vaccine.2014.11.013 25448107

[28] Nambiar JK Pinto R Aguilo JI Takatsu K Martin C Britton WJ Protective immunity afforded by attenuated, PhoP-deficient *Mycobacterium tuberculosis* is associated with sustained generation of CD4+ T-cell memory. Eur J Immunol (2012) 42:385–92. 10.1002/eji.201141903 22105536

[29] Andersen P Woodworth JS Tuberculosis vaccines – rethinking the current paradigm. Trends Immunol (2014) 35:387–95. 10.1016/j.it.2014.04.006 24875637

[30] Luabeya AK Kagina BM Tameris MD Geldenhuys H Hoff ST Shi Z First-in-human trial of the post-exposure tuberculosis vaccine H56:IC31 in *Mycobacterium tuberculosis* infected and non-infected healthy adults. Vaccine (2015) 33:4130–40. 10.1016/j.vaccine.2015.06.051 26095509

[31] Tameris M Hokey DA Nduba V Sacarlal J Laher F Kiringa G A double-blind, randomised, placebo-controlled, dose-finding trial of the novel tuberculosis vaccine AERAS-402, an adenovirus-vectored fusion protein, in healthy, BCG-vaccinated infants. Vaccine (2015) 33:2944–54. 10.1016/j.vaccine.2015.03.070 25936724 PMC6698638

[32] Lindenstrøm T Knudsen NP Agger EM Andersen P. Control of chronic *Mycobacterium tuberculosis* infection by CD4 KLRG1- IL-2-secreting central memory cells. J Immunol (2013) 190:6311–9. 10.4049/jimmunol.1300248 23677471

[33] Kagina BM Abel B Scriba TJ Hughes EJ Keyser A Soares A Specific T cell frequency and cytokine expression profile do not correlate with protection against tuberculosis after bacillus Calmette-Guérin vaccination of newborns. Am J Respir Crit Care Med (2010) 182(8):1073–9. 10.1164/rccm.201003-0334OC 20558627 PMC2970848

[34] Adekambi T Ibegbu CC Kalokhe AS Yu T Ray SM Rengarajan J. Distinct effector memory CD4+ T cell signatures in latent *Mycobacterium tuberculosis* infection, BCG vaccination and clinically resolved tuberculosis. PLoS One (2012) 7:e36046. 10.1371/journal.pone.0036046 22545156 PMC3335801

[35] Dintwe OB Day CL Smit E Nemes E Gray C Tameris M Heterologous vaccination against human tuberculosis modulates antigen-specific CD4+ T-cell function. Eur J Immunol (2013) 43:2409–20. 10.1002/eji.201343454 23737382 PMC3816254

[36] Dean GS Whelan A Clifford D Salguero FJ Xing Z Gilbert S Comparison of the immunogenicity and protection against bovine tuberculosis following immunization by BCG-priming and boosting with adenovirus or protein based vaccines. Vaccine (2015) 32:1304–10. 10.1016/j.vaccine.2013.11.045 24269321

[37] Behr MA Wilson MA Gill WP Salamon H Schoolnik GK Rane S Comparative genomics of BCG vaccines by whole-genome DNA microarray. Science (1999) 284:1520–3. 10.1126/science.284.5419.1520 10348738

[38] Van Pittius NCG Gamieldien J Hide W Brown GD Siezen RJ Beyers AD. The ESAT-6 gene cluster of *Mycobacterium tuberculosis* and other high G+C Gram-positive bacteria. Genome Biol (2001) 2(10):RESEARCH0044. 11597336 10.1186/gb-2001-2-10-research0044 PMC57799

[39] Mahairas GG Sabo PJ Hickey MJ Singh DC Stover CK. Molecular analysis of genetic differences between *Mycobacterium bovis* BCG and virulent *M. bovis*. J Bacteriol (1996) 178:1274–82. 8631702 10.1128/jb.178.5.1274-1282.1996 PMC177799

[40] Pym AS Brodin P Brosch R Huerre M Cole ST. Loss of RD1 contributed to the attenuation of the live tuberculosis vaccines *Mycobacterium bovis* BCG and *Mycobacterium microti*. Mol Microbiol (2002) 46:709–17. 10.1046/j.1365-2958.2002.03237.x 12410828

[41] Buddle BM Parlane NA Keen DL Aldwell FE Pollock JM Lightbody K Differentiation between *Mycobacterium bovis* BCG-vaccinated and *M. bovis*-infected cattle by using recombinant mycobacterial antigens. Clin Diagn Lab Immunol (1999) 6:1–5. 9874655 10.1128/cdli.6.1.1-5.1999 PMC95651

[42] Pollock JM Andersen P The potential of the ESAT-6 antigen secreted by virulent mycobacteria for specific diagnosis of tuberculosis. Infect Immun (2000) 1997(175):2587–92. 10.1086/593686 9129098

[43] van Pinxteren LA Ravn P Agger EM Pollock J Andersen P. Diagnosis of tuberculosis based on the two specific antigens ESAT-6 and CFP10. Clin Diagn Lab Immunol (2000) 7:155–60. 10.1128/CDLI.7.2.155-160.2000 10702486 PMC95842

[44] Vordermeier HM Whelan A Cockle PJ Farrant L Palmer N Hewinson RG. Use of synthetic peptides derived from the antigens ESAT-6 and CFP-10 for differential diagnosis of bovine tuberculosis in cattle. Clin Diagn Lab Immunol (2001) 8:571–8. 10.1128/CDLI.8.3.571-578.2001 11329460 PMC96103

[45] Waters WR Nonnecke BJ Palmer MV Robbe-Austermann S Bannantine JP Stabel JR Use of recombinant ESAT-6:CFP-10 fusion protein for differentiation of infections of cattle by *Mycobacterium bovis* and by *M. avium* subsp. *avium* and *M. avium* subsp. *paratuberculosis*. Clin Diagn Lab Immunol (2004) 11:729–35. 10.1128/CDLI.11.4.729-735.2004 15242948 PMC440606

[46] Wedlock DN Denis M Vordermeier HM Hewinson RG Buddle BM. Vaccination of cattle with Danish and Pasteur strains of *Mycobacterium bovis* BCG induce different levels of IFN gamma post-vaccination, but induce similar levels of protection against bovine tuberculosis. Vet Immunol Immunopathol (2007) 118:50–8. 10.1016/j.vetimm.2007.04.005 17524495

[47] Buddle BM Hewinson RG Vordermeier HM Wedlock DN Subcutaneous administration of a 10-fold-lower dose of a commercial human tuberculosis vaccine, *Mycobacterium bovis* bacillus Calmette-Guerin Danish, induced levels of protection against bovine tuberculosis and responses in the tuberculin intradermal test similar to those induced by a standard cattle dose. Clin Vaccine Immunol (2013) 20:1559–62. 10.1128/CVI.00435-13 23925885 PMC3807190

[48] Dao DN Sweeney K Hsu T Gurcha SS Nascimento IP Roshevsky D Mycolic acid modification by the mmaA4 gene of *M. tuberculosis* modulates IL-12 production. PLoS Pathog (2008) 4:e1000081. 10.1371/journal.ppat.1000081 18535659 PMC2390761

[49] Berney M Berney-Meyer L Wong KW Chen B Chen M Kim J Essential roles of methionine and S-adenosylmethionine in the autarkic lifestyle of Mycobacterium tuberculosis. Proc Natl Acad Sci U S A (2015) 112:10008–13. 10.1073/pnas.1513033112 26221021 PMC4538671

[50] Panas MW Sixsmith JD White K Korioth-Schmitz B Shields ST Moy BT Gene deletions in *Mycobacterium bovis* BCG stimulate increased CD8+ T cell responses. Infect Immun (2014) 82:5317–26. 10.1128/IAI.02100-14 25287928 PMC4249275

[51] Waters WR Maggioli MF Palmer MV Thacker TC McGill JL Vordermeier HM Interleukin-17A as a biomarker for bovine tuberculosis. Clin Vaccine Immunol (2015) 23:168–80. 10.1128/CVI.00637-15 26677202 PMC4744917

[52] Palmer MV Waters WR Whipple DL. Aerosol delivery of virulent *Mycobacterium bovis* to cattle. Tuberculosis (2002) 82:275–82. 10.1054/tube.2002.0341 12623270

[53] Vordermeier HM Chambers MA Cockle PJ Whelan AO Simmons J Hewinson RG. Correlation of ESAT-6-specific gamma interferon production with pathology in cattle following *Mycobacterium bovis* BCG vaccination against experimental bovine tuberculosis. Infect Immun (2002) 70:3026–32. 10.1128/IAI.70.6.3026-3032.2002 12010994 PMC128013

[54] Wangoo A Johnson L Gough J Ackbar R Inglut S Hicks D Advanced granulomatous lesions in *Mycobacterium bovis*-infected cattle are associated with increased expression of type I procollagen, gammadelta (WC1+) T cells and CD 68+ cells. J Comp Pathol (2005) 133:223–34. 10.1016/j.jcpa.2005.05.001 16154140

[55] Aabye MG Ravn P Johansen IS Eugen-Olsen J Ruhwald M Incubation of whole blood at 39°C augments gamma interferon (IFN-γ)-induced protein 10 and IFN-γ responses to *Mycobacterium tuberculosis* antigens. Clin Vaccine Immunol (2011) 18:1150–6. 10.1128/CVI.00051-11 21613464 PMC3147319

[56] Maggioli MF Palmer MV Vordermeier HM Whelan AO Fosse JM Nonnecke BJ Application of long-term cultured interferon-γ enzyme-linked immunospot assay for assessing effector and memory T cell responses in cattle. J Vis Exp (2015) 101:e52833. 10.3791/52833 26275095 PMC4544920

[57] Palmer MV Waters WR Thacker TC. Lesion development and immunohistochemical changes in granulomas from cattle experimentally infected with *Mycobacterium bovis*. Vet Pathol (2007) 44:863–74. 10.1354/vp.44-6-863 18039899

[58] Waters WR Palmer MV Nonnecke BJ Thacker TC Scherer CF Estes DM Failure of a *Mycobacterium tuberculosis* δRD1 δpanCD double deletion mutant in a neonatal aerosol *M. bovis* challenge model: comparisons to responses elicited by *M. bovis* bacilli Calmette Guerin. Vaccine (2007) 2545:7832–40. 10.1016/j.vaccine.2007.08.029 17931755

[59] Diedrich CR Flynn JL. HIV-1/*Mycobacterium tuberculosis* coinfection immunology: how does HIV-1 exacerbate tuberculosis? Infect Immun (2011) 79:1407–17. 10.1128/IAI.01126-10 21245275 PMC3067569

[60] Campion S Cohen MS McMichael AJ Galvin S Goonetilleke N Improved detection of latent *Mycobacterium tuberculosis* infection in HIV-1 seropositive individuals using cultured cellular assays. Eur J Immunol (2011) 41:255–7. 10.1002/eji.201040296 21182097 PMC3119189

[61] Calarota SA Baldanti F. Enumeration and characterization of human memory T cells by enzyme-linked immunospot assays. Clin Dev Immunol (2013) 2013:637649. 10.1155/2013/637649 24319467 PMC3844203

[62] Calarota SA Foli A Maserati R Baldanti F Baldanti F Paolucci S HIV-1-specific T cell precursors with high proliferative capacity correlate with low viremia and high CD4 counts in untreated individuals. J Immunol (2008) 180:5907–15. 10.4049/jimmunol.180.9.5907 18424710

[63] Calarota SA Chiesa A Zelini P Comolli G Minoli L Baldanti F Detection of Epstein-Barr virus-specific memory CD4+ T cells using a peptide-based cultured enzyme-linked immunospot assay. Immunology (2012) 139:533–44. 10.1111/imm.12106 PMC3719070 23560877

[64] Pinder M Reece WH Plebanski M Akinwunmi P Flanagan KL Lee EA Cellular immunity induced by the recombinant *Plasmodium falciparum* malaria vaccine, RTS,S/AS02, in semi-immune adults in The Gambia. Clin Exp Immunol (2004) 135:286–93. 10.1111/j.1365-2249.2004.02371.x 14738458 PMC1808944

[65] Todryk SM Pathan AA Keating S Porter DW Berthoud T Thompson F The relationship between human effector and memory T cells measured by *ex vivo* and cultured ELISPOT following recent and distal priming. Immunology (2009) 128:83–91. 10.1111/j.1365-2567.2009.03073.x 19689738 PMC2747141

[66] Moser JM Sassano ER Leistritz del C Eatrides JM Phogat S Koff W Optimization of a dendritic cell-based assay for the in vitro priming of naïve human CD4+ T cells. J Immunol Methods (2010) 353:8–19. 10.1016/j.jim.2009.11.006 19925804

[67] Chang JT Palanivel VR Kinjyo I Schambach F Intlekofer AM Banerjee A Asymmetric T lymphocyte division in the initiation of adaptive immune responses. Science (2007) 315:1687–91. 10.1126/science.1139393 17332376

[68] Laouar A Manocha M Haridas V Manjunath N. Concurrent generation of effector and central memory CD8 T cells during vaccinia virus infection. PLoS One (2008) 3:e4089. 10.1371/journal.pone.0004089 19116651 PMC2605255

[69] Teixeiro E Daniels MA Hamilton SE Schrum AG Bragado R Jameson SC Different T cell receptor signals determine CD8+ memory versus effector development. Science (2009) 323:502–5. 10.1126/science.1163612 19164748

[70] King CG Koehli S Hausmann B Schmaler M Zehn D Palmer E. T cell affinity regulates asymmetric division, effector cell differentiation, and tissue pathology. Immunity (2012) 37:709–20. 10.1016/j.immuni.2012.06.021 23084359 PMC3622938

[71] Tubo NJ Pagan AJ Taylor JJ Nelson RW Linehan JL Ertelt JM Single naive CD4+ T cells from a diverse repertoire produce different effector cell types during infection. Cell (2013) 153:785–96. 10.1016/j.cell.2013.04.007 23663778 PMC3766899

[72] Tubo NJ Fife BT Pagan AJ Kotov DI Goldberg MF Jenkins MK. Most microbe-specific naïve CD4^+^ T cells produce memory cells during infection. Science (2016) 351:511–4. 10.1126/science.aad0483 26823430 PMC4776317

[73] Swain SL Generation and *in vivo* persistence of polarized 1 and 2 memory cells. Immunity (1994) 1:543–52. 10.1016/1074-7613(94)90044-2 7600283

[74] Swain SL Hu H Huston G. Class II-independent generation of CD4 memory T cells from effectors. Science (1999) 28:1381–3. 10.1126/science.286.5443.1381 10558997

[75] Pepper M Jenkins MK. Origins of CD4(+) effector and central memory T cells. Nat Immunol (2011) 12:467–71. 10.1038/ni.2038 21739668 PMC4212218

[76] Moulton VR Bushar ND Leeser DB Patke DS Farber DL. Divergent generation of heterogeneous memory CD4 T cells. J Immunol (2006) 177:869–76. 10.4049/jimmunol.177.2.869 16818741

[77] Lohning M Hegazy AN Pinschewer DD Busse D Lang KS Höfer T Long-lived virus-reactive memory T cells generated from purified cytokine-secreting T helper type 1 and type 2 effectors. J Exp Med (2008) 205:53–61. 10.1084/jem.20071855 18195073 PMC2234365

[78] Opata MM Stephens R. Early decision: effector and effector memory T cell differentiation in chronic infection. Curr Immunol Rev (2013) 9:190–206. 10.2174/1573395509666131126231209 24790593 PMC4000274

[79] Gasper DJ Tejera MM Suresh M. CD4 T-cell memory generation and maintenance. Crit Rev Immunol (2014) 34:121–46. 10.1615/CritRevImmunol.2014010373 24940912 PMC4062920

[80] Reiley WW Shafiani S Wittmer ST Tucker-Heard G Moon JJ Jenkins MK Distinct functions of antigen-specific CD4 T cells during murine *Mycobacterium tuberculosis* infection. Proc Natl Acad Sci U S A (2010) 107:19408–13. 10.1073/pnas.1006298107 20962277 PMC2984157

[81] Harari A Rozot V Bellutti Enders F Perreau M Stalder JM Nicod LP Dominant TNF-α+ *Mycobacterium tuberculosis*-specific CD4+ T cell responses discriminate between latent infection and active disease. Nat Med (2011) 17:372–6. 10.1038/nm.2299 21336285 PMC6570988

[82] Petruccioli E Petrone L Vanini V Sampaolesi A Gualano G Girardi E IFNγ/TNFα specific-cells and effector memory phenotype associate with active tuberculosis. J Infect (2013) 66:475–86. 10.1016/j.jinf.2013.02.004 23462597

[83] Ziraldo C Gong C Kirschner DE Linderman JJ. Strategic priming with multiple antigens can yield memory cell phenotypes optimized for Infection with *Mycobacterium tuberculosis*: a computational study. Front Microbiol (2016) 6:1477. 10.3389/fmicb.2015.01477 26779136 PMC4701940

[84] Sakai S Kauffman KD Schenkel JM McBerry CC Mayer-Barber KD Masopust D Cutting edge: control of *Mycobacterium tuberculosis* infection by a subset of lung parenchyma-homing CD4 T cells. J Immunol (2014) 192:2965–9. 10.4049/jimmunol.1400019 24591367 PMC4010124

[85] Kaushal D Foreman TW Gautam US Alvarez X Adekambi T Rangel-Moreno J Mucosal vaccination with attenuated *Mycobacterium tuberculosis* induces strong central memory responses and protects against tuberculosis. Nat Commun (2015) 6:8533. 10.1038/ncomms9533 26460802 PMC4608260

