# Supplementary material for: Increased TNF-α/IFN-γ/IL-2 and Decreased TNF-α/IFN-γ Production by Central Memory T Cells Are Associated with Protective Responses against Bovine Tuberculosis Following BCG Vaccination
Source: Front Immunol. 2016 Oct 17;7:421. doi: 10.3389/fimmu.2016.00421 (PMC5066095; doi:10.3389/fimmu.2016.00421)
Supplement: Supplementary file 1 [file presentation_1.pdf]

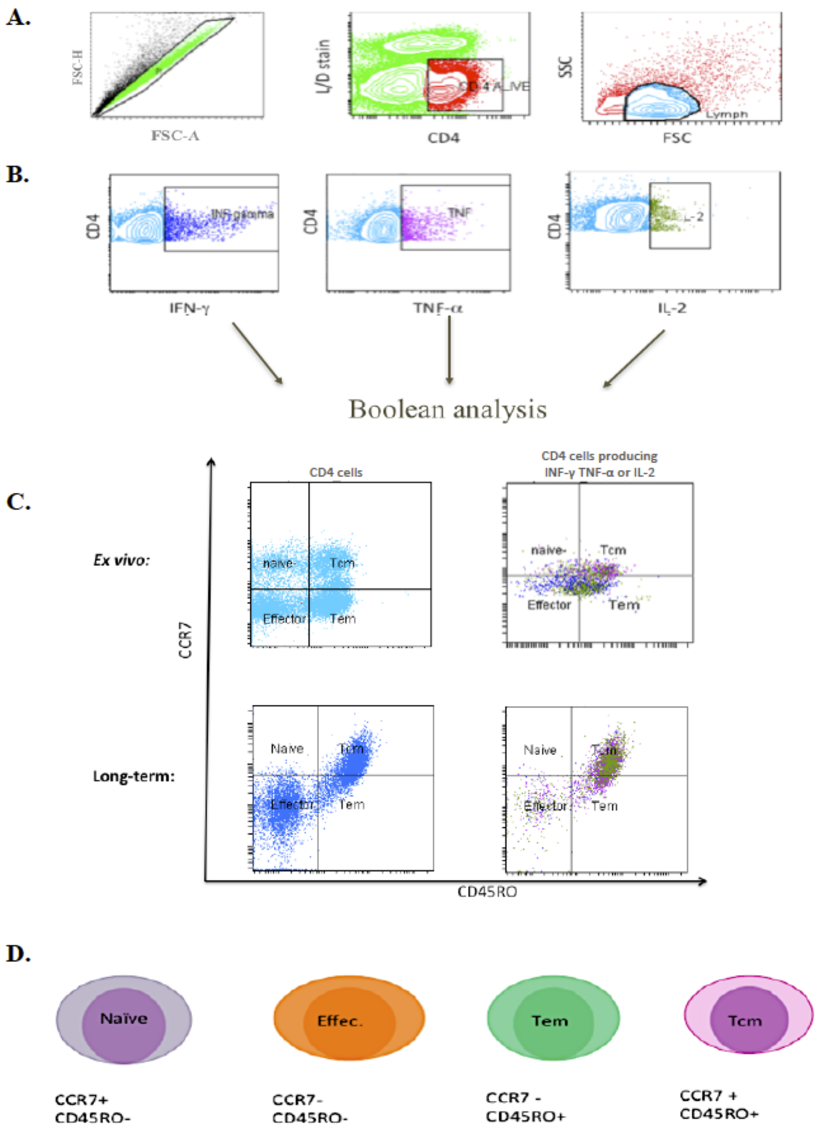

S1. Gating strategy for assessment of polyfunctional cytokine responses by effector/memory phenotypes in long-term and *ex vivo* culture conditions. A representative polyfunctional response from an infected animal is presented to demonstrate the gating strategy. (**A**) Selection of populations containing live $CD4^+$ singlets within the lymphocyte gate (based on FSC and SSC properties). Each subsequent panel shows only the population of interest that has been selected from the gate on the previous plot. (**B**) CD4 cells producing IFN-γ, IL-2 or TNF-α in response to PPD, followed by combinatory Boolean analysis for polyfunctional cytokine profiles. (**C**) CD4 T cells producing any cytokines of interest were then analyzed for CD45RO/CCR7 expression to further discriminate the polyfunctional CD4 T cell response by effector/memory phenotype in *ex vivo* (light blue) and long-term cultures (dark blue). (**D**) Expression of cell markers on different T cell memory/effector subsets.

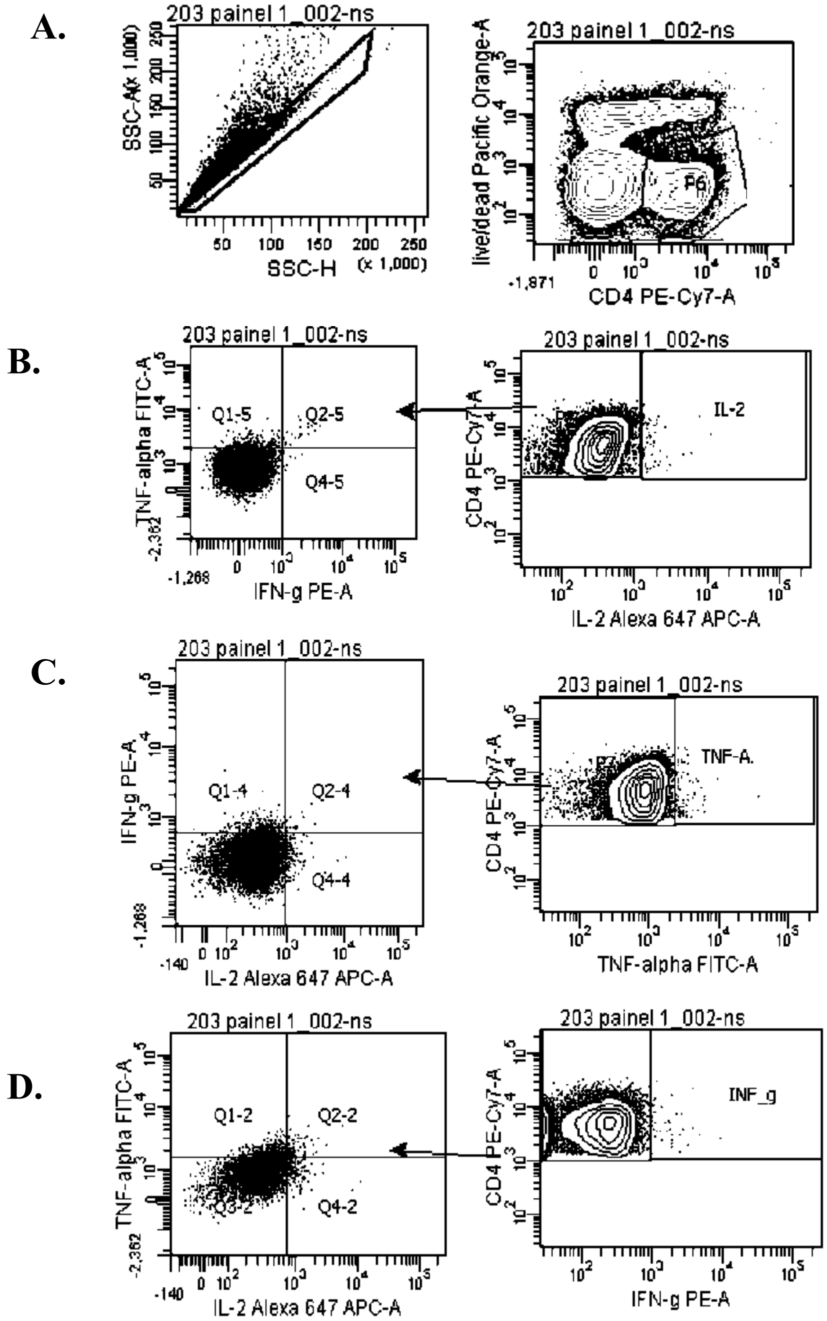

S2. Cytokine production under unstimulated condition. A representative polyfunctional response from an infected animal is presented. (**A**) Selection of populations containing live $CD4^+$ singlets within the lymphocyte gate (based on FSC and SSC properties). Each subsequent panel shows only the population of interest that has been selected from the gate on the previous plot. CD4 cells producing (B) IL-2, (C) TNF-α or (D) IFN-γ (C) in the absence of antigenic stimulation. Subgating (left side plots) of $CD4^+$ show all possible functionalities for the expression of the 3 cytokines.

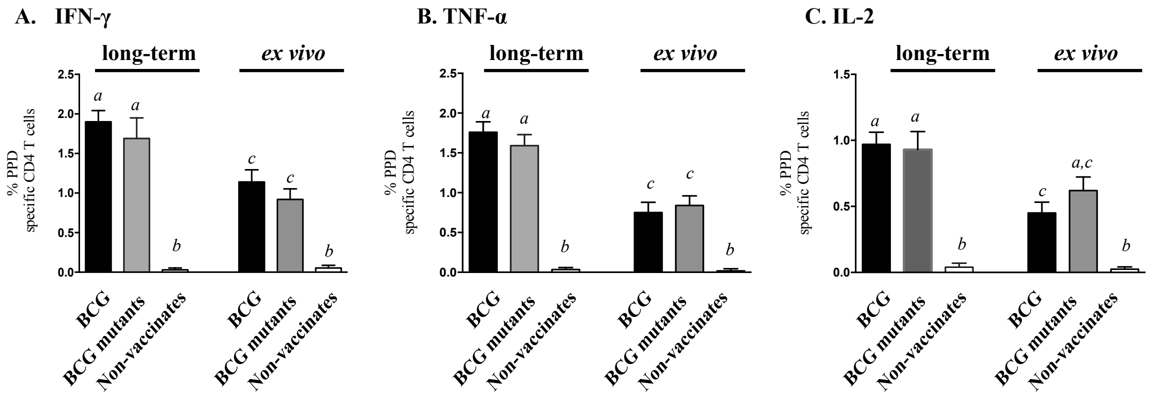

S3 . Cytokine production by CD4 T cells under long-term or *ex vivo* culture conditions in response to vaccination. For long-term culture, PBMCs were isolated and stimulated with a cocktail of Ag85A, TB10.4, and PPD for 13 days followed by transfer to 96 well round bottom plates with APCs and addition of media alone or PPD for an additional 16h. For *ex vivo* cultures, PBMCs were isolated and stimulated with media alone or PPD (5 μg/ml) for 16h. Results are presented as average and standard error. Frequency of long-term and *ex vivo* cultured CD4 T cells producing IFN-γ (**A**), TNF-α (**B**), or IL-2 (**C**).

[abc]Different letters represent differences ($p < 0.05$) in cytokine production in long-term and *ex vivo* culture conditions for each individual cytokine (ANOVA followed by Tukey's multiple comparisons test).

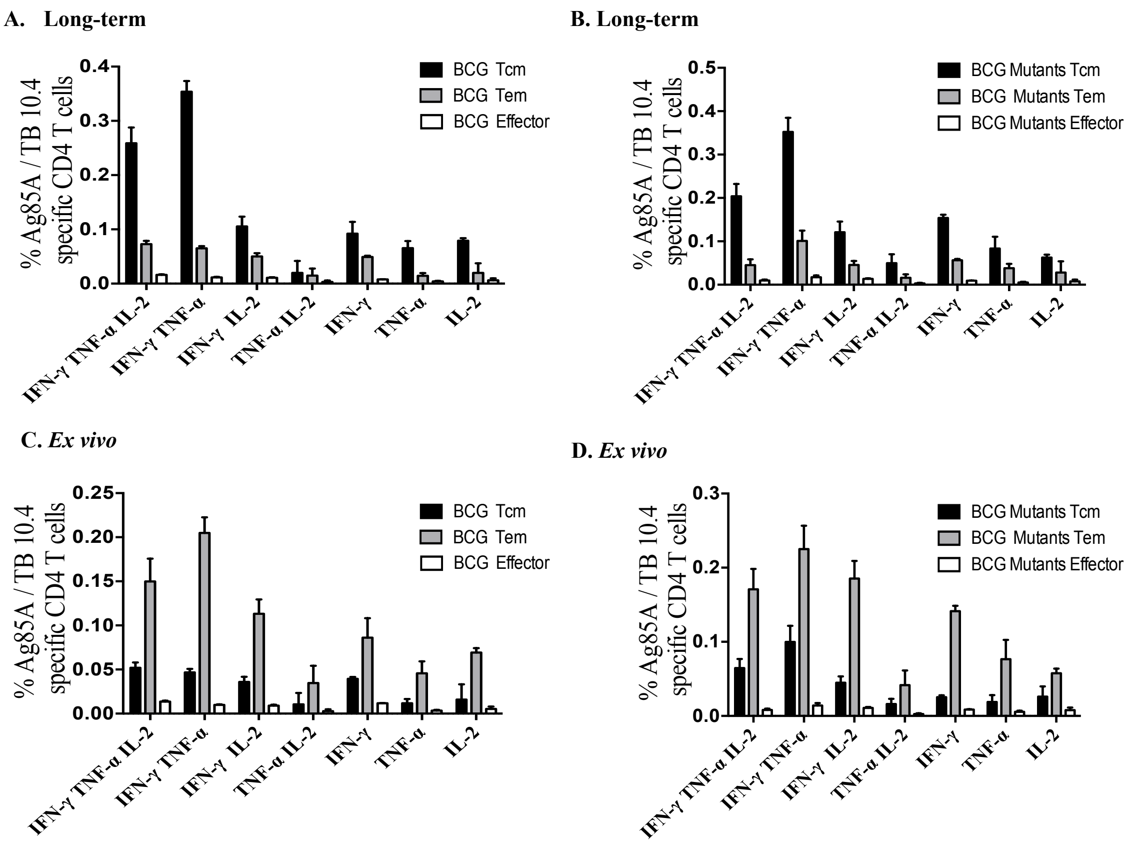

S4. Polyfunctional cytokine production to Ag85A/TB10.4 by PBMCs from BCG or BCG mutants under long-term or *ex vivo* cultures at 6 weeks post vaccination. For long-term culture, PBMCs were isolated and stimulated with a cocktail of Ag85A, TB10.4, and PPD for 13 days followed by transfer to 96 well round bottom plates with APCs and addition of media alone or Ag85A/TB10.4 for an additional 16h. For *ex vivo* culture, PBMCs were stimulated with media alone or a cocktail of Ag85A/TB10.4 for 16h. Results are presented as average and standard error. Percentage of cytokine production profiles among memory subsets in response to recall stimulation of long-term cultures with Ag85A/TB10.4 by BCG-vaccinates (**A**) or BCG mutants-vaccinates (**B**). Percentage of the cytokine production profiles among memory subsets in response to *ex vivo* stimulation with Ag85A/TB10.4 by BCG-vaccinates (**C**) or BCG mutants-vaccinates (**D**).

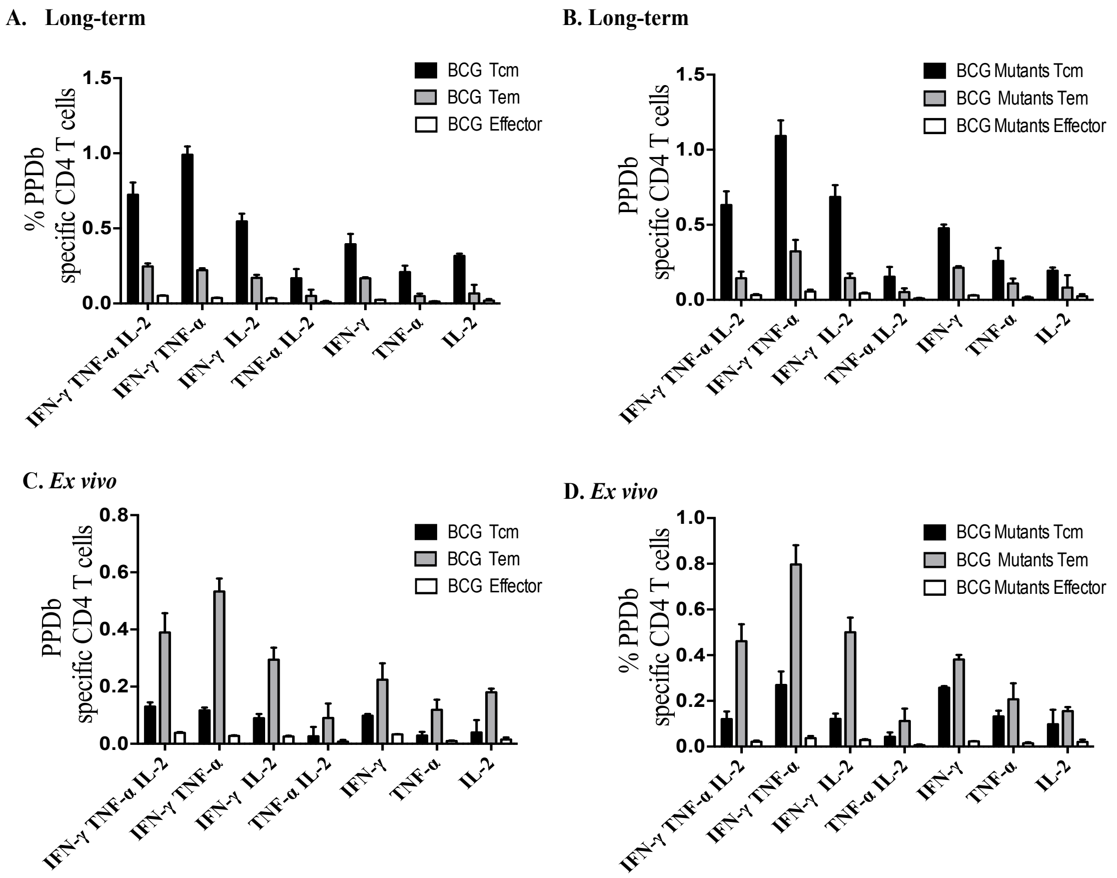

S5. Polyfunctional cytokine production to PPD by PBMCs from BCG mutants- or BCG-vaccinates under long-term or *ex vivo* cultures at 6 weeks post vaccination. For long-term culture, PBMCs were isolated and stimulated with a cocktail of Ag85A, TB10.4, and PPD for 13 days followed by transfer to 96 well round bottom plates with APCs and addition of media alone or PPD for an additional 16h. For *ex vivo* culture, PBMCs were stimulated with media alone or PPD for 16 h. Results are presented as average and standard error. Percentage of the cytokine production profiles among memory subsets in response to recall stimulation of long-term cultures with PPD by BCG-vaccinates (**A**) or BCG mutants-vaccinates (**B**). Percentage of the cytokine production profiles among memory subsets in response to *ex vivo* stimulation with PPD by BCG-vaccinates (**C**) or BCG mutants-vaccinates (**D**).

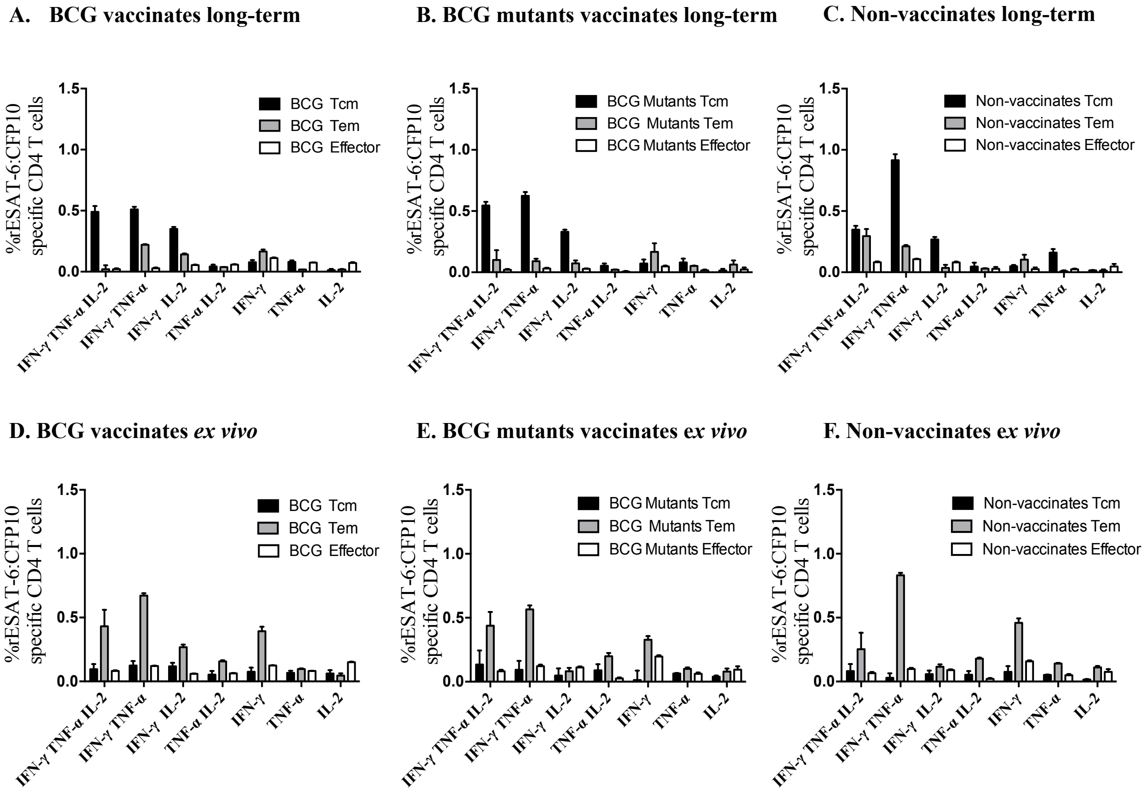

S6. Polyfunctional cytokine production by PBMCs from BCG mutants or BCG vaccinates under long-term or *ex vivo* culture conditions. For long-term culture, PBMCs were isolated at 3 weeks post infection and stimulated with a cocktail of rAg85A, rTB10.4, and PPD for 13 days followed by transfer to 96 well round bottom plates with APCs and addition of media alone or ESAT-6:CFP10 for an additional 16h. For *ex vivo* culture, PBMCs were stimulated with media alone or ESAT-6:CFP10 for 16 h. Results are presented as average and standard error. Percentage of the cytokine production profiles among memory subsets in response to recall stimulation of long-term cultures with ESAT-6:CFP10 by BCG-vaccinates (**A**), BCG mutants-vaccinates (**B**) or non-vaccinates (**C**). Percentage of the cytokine production profiles among memory subsets in response to *ex vivo* stimulation with ESAT-6:CFP10 by BCG-vaccinates (**D**), BCG mutants-vaccinates (**E**) or non-vaccinates (**F**).

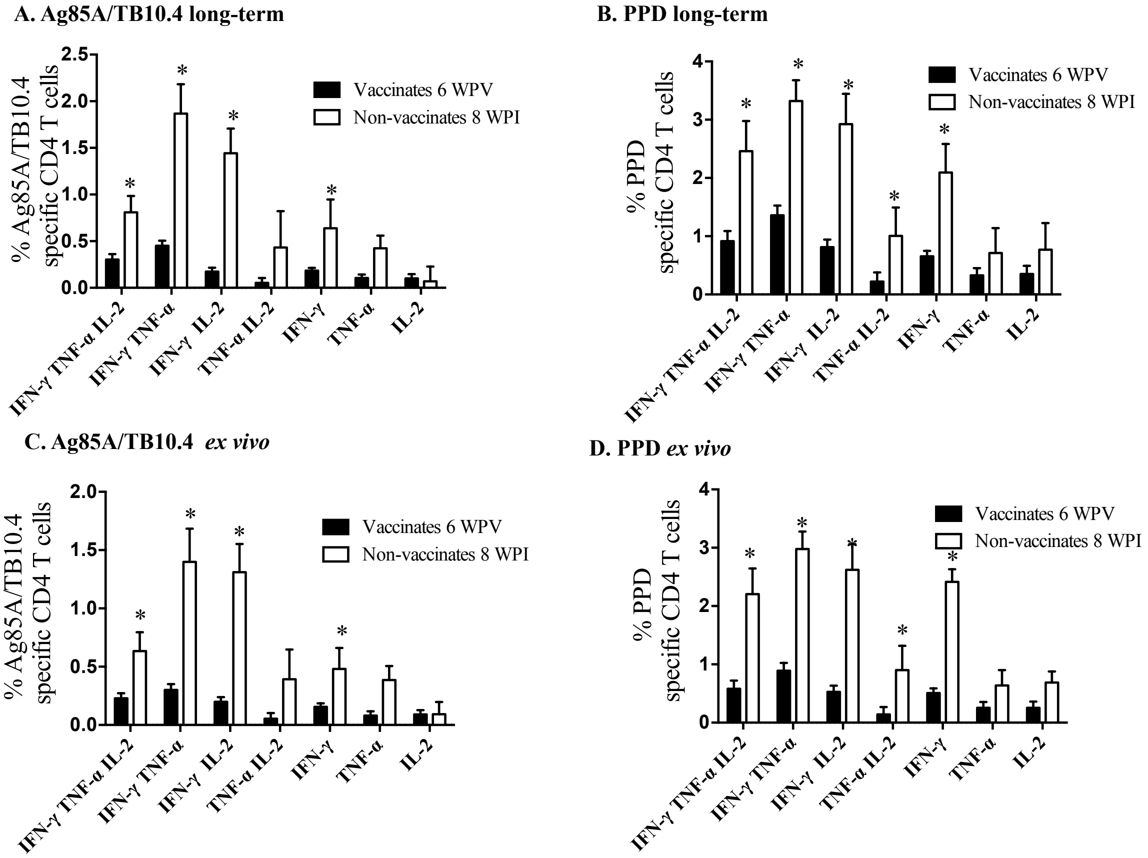

S7. Cytokine production elicited by *M. bovis* infection exceeded respective responses to vaccination. For long-term culture, PBMCs were isolated at 8 weeks post infection and stimulated with a cocktail of rAg85A, rTB10.4, and PPD for 13 days followed by transfer to 96 well round bottom plates with APCs and addition of media alone or ESAT-6:CFP10 for an additional 16h. For *ex vivo* culture, PBMCs were stimulated with media alone or ESAT-6:CFP10 for 16 h. Results are presented as average and standard error. Percentage of the cells exhibiting polyfunctional profile to vaccination (6 WPV) and infection (non-vaccinates; 8WPI), in response to recall stimulation of long-term cultures with Ag85A/TB10.4 (**A**) or PPD (**B**). Percentage of the cells exhibiting polufunctional profile to vaccination (6 WPV) and infection (non-vaccinates; 8WPI), in response *ex vivo* recall stimulation with Ag85A/TB10.4 (**C**) or PPD (**D**).
* Differences ($P < 0.05$, Tukey's) in the cytokine production between by vaccinates (at 6WPV) and non-vaccinates at either 3 or 8 WPI (ANOVA followed by Tukey's multiple comparisons test).
